# Disruption of microbial community composition and identification of plant growth promoting microorganisms after exposure of soil to rapeseed-derived glucosinolates

**DOI:** 10.1371/journal.pone.0200160

**Published:** 2018-07-03

**Authors:** Meike Siebers, Thomas Rohr, Marina Ventura, Vadim Schütz, Stephan Thies, Filip Kovacic, Karl-Erich Jaeger, Martin Berg, Peter Dörmann, Margot Schulz

**Affiliations:** 1 Institute of Molecular Physiology and Biotechnology of Plants (IMBIO), University of Bonn, Bonn, Germany; 2 Institute of Molecular Enzyme Technology, Heinrich Heine University Düsseldorf, Forschungszentrum Jülich, Jülich, Germany; 3 Institute of Bio- and Geosciences IBG-1: Biotechnology, Forschungszentrum Jülich, Jülich, Germany; 4 Institute for Organic Agriculture, University of Bonn, Bonn, Germany; 5 Experimental Farm Wiesengut of University of Bonn, Hennef, Germany; Estacion Experimental del Zaidin, SPAIN

## Abstract

Land plants are engaged in intricate communities with soil bacteria and fungi indispensable for plant survival and growth. The plant-microbial interactions are largely governed by specific metabolites. We employed a combination of lipid-fingerprinting, enzyme activity assays, high-throughput DNA sequencing and isolation of cultivable microorganisms to uncover the dynamics of the bacterial and fungal community structures in the soil after exposure to isothiocyanates (ITC) obtained from rapeseed glucosinolates. Rapeseed-derived ITCs, including the cyclic, stable goitrin, are secondary metabolites with strong allelopathic affects against other plants, fungi and nematodes, and in addition can represent a health risk for human and animals. However, the effects of ITC application on the different bacterial and fungal organisms in soil are not known in detail. ITCs diminished the diversity of bacteria and fungi. After exposure, only few bacterial taxa of the Gammaproteobacteria, Bacteriodetes and Acidobacteria proliferated while *Trichosporon* (Zygomycota) dominated the fungal soil community. Many surviving microorganisms in ITC-treated soil where previously shown to harbor plant growth promoting properties. Cultivable fungi and bacteria were isolated from treated soils. A large number of cultivable microbial strains was capable of mobilizing soluble phosphate from insoluble calcium phosphate, and their application to *Arabidopsis* plants resulted in increased biomass production, thus revealing growth promoting activities. Therefore, inclusion of rapeseed-derived glucosinolates during biofumigation causes losses of microbiota, but also results in enrichment with ITC-tolerant plant microorganisms, a number of which show growth promoting activities, suggesting that Brassicaceae plants can shape soil microbiota community structure favoring bacteria and fungi beneficial for *Brassica* plants.

## Introduction

Glucosinolates (GSLs) are secondary metabolites of the Brassicales, a plant order including the family of Brassicaceae with numerous crop species, e.g. *Brassica napus* (rapeseed, canola), *Brassica oleracea* (vegetables) and others. Rapeseed belongs to the most important oil crops, with Europe, Canada and China representing the leading production areas. GSLs are stored in plant cells and only after tissue damage, the compounds come in contact with thioglucosidases (myrosinases). Hydrolysis of GSLs yields glucose and an aglycone, a precursor for mainly volatile isothiocyanates (ITCs) with strong biocidal activity [1]. Although characterized by a short half-life (from hours to days), ITCs exert strong allelopathic effects against many plants, fungi and nematodes. In contrast, cyclized 2-hydroxy-3-butenyl ITC (goitrin) is a stable, water soluble ITC derived from progoitrin, the major GSL in rapeseed and other *Brassica* species. Goitrin represents a health risk because of the goitrogenic properties, i.e. the inhibition of iodine uptake into the thyroid gland which can result in goiter formation. The introduction of GSL-producing plants can have a dramatic impact on natural ecosystems. For example, garlic mustard (*Alliaria petiolata*), another Brassicaceae species and an aggressive invader in the USA, succeeded in the destruction of beneficial fungi living in a mutualistic lifestyle with native trees by ITC allelopathy [2,3].

The aboveground plant organs have been the main target for studying GSLs [4]. Breeding efforts in the past 50 years led to a strong reduction of the GSL content in modern canola varieties [5]. However, even elite canola cultivars contain considerable amounts of GSLs in seeds and roots [4]. Because spring and winter varieties of rapeseed grow about 6 or 12 months until harvest, respectively, considerable amounts of GSL are introduced into the soil [6]. Seeds of *Brassica napus* contain 10–100 mmol kg^-1^ of total GSLs [7]. Taking into account the recommended sowing quantity for canola (~ 5 kg ha^-1^), 50–500 mmol ha^-1^ of GSL are released during germination. In addition, GSL biosynthesis is induced by wounding, plant diseases and hormones, and also depends on the genetic background of the cultivar. Therefore, it is challenging to estimate the total amount of GSL introduced into the soil under field conditions [8].

*Brassica napus* has been recommended as a rotational cover crop for biofumigation, but this causes the accumulation of ITCs in the soil, affecting growth of subsequent crops. The effects of the stable ITC goitrin might be long lasting. Thus, monocultured rapeseed shows a 25% yield reduction in shortened crop rotation [9], and rapeseed causes growth suppression during subsequent sunflower cultivation [10].

The repercussions of ITCs on fungi have previously been reported [11], with research focused on soil-borne fungal pathogens. Bacteria of the Rhizobiaceae and Alphaproteobacteria were particularly compromised by ITCs [12], but it is believed that bacteria are less affected than fungi. Only few studies focused on the long-term effects of ITCs on the proliferation of microorganisms and the shift in microbial diversity [13–15]. Application of GSLs affects the soil microbial community by favoring *Brassica*-compatible fungi and bacteria [12]. GSLs exert a considerable impact on fungal rhizosphere communities, accompanied with a decline in diversity, but with an enhanced growth of pathogenic fungi [9]. The operational taxonomic unit (OTU)-based biodiversity of fungi dropped to less than 60% after mustard seed meal application [14], and a reduction of 85% of fungal species was reported after application of allyl-ITC to the soil [13].

Loss of fungal diversity is certainly not the only consequence of GSL release into the soil. Plant growth enhancement by fungal volatile compounds includes the induction of systemic resistance and abiotic stress tolerance [16]. For example, *Fusarium oxysporum* strains harboring ectosymbiotic bacteria promote shoot growth of plants by volatile-dependent manipulation of auxin transport and signaling. Rapeseed cultivation or biofumigation might result in their elimination.

Previous studies on the effects of GSL on soil microorganisms employing DNA sequencing approaches revealed a general decrease in microbial diversity. However, details on the impact of GSL on biodiversity and on the functional aspects of surviving soil bacteria and fungi remained unclear. Furthermore, it is conceivable that GLS treatment will cause the death of soil microbiota. DNA sequencing of PCR amplicons cannot easily distinguish between live and dead biomass, because specific pretreatments, e.g. with propidium monoazide, are required to remove extracellular ("relic") DNA prior to sequencing [17]. Here, we present a comprehensive study including high-throughput lipidomics, enzyme measurements, and genomics analyses to unravel the manifold effects of GSL on soil microorganisms. Using this approach, it was possible to demonstrate the dying of microorganisms, and we provide evidence that the surviving microbiota contains several plant growth promoting strains.

## Materials and methods

### Soil and microcosm

Fresh samples of an organic farming, species-rich, loamy sand (sU to sIU) (pH 6.1; ~1.4% organic matter, 16–18% moisture upon sampling), were collected from the upper 30 cm (A-horizon) of a field at Wiesengut (Siegaue, Hennef, Germany; geographical position 65 m above NN; 7° 17' East; 50° 48' North; www.wiesengut.uni-bonn.de). The teaching and testing site for organic farming "Wiesengut" is an experimental station of University of Bonn. Therefore, no specific permission is required for members of the University of Bonn. No endangered or protected species were involved in this study.

The field had been covered with spring wheat (2010), followed by winter wheat (2011), red clover (2012), and again spring wheat (2013). Before sampling in March 2015, the field was cultivated with winter wheat and Persian clover as catch crop ensuring that soil was not affected by GSLs or herbicides. After sampling, the soil was air dried (< 14 d, room temperature), sieved (2 mm mesh) and stored in plastic bags at –20°C. The soil was characterized by Raiffeisen-Laborservice (Omont, Germany) for texture, pH, organic matter, and nutrient contents (S1 Table).

### Extraction of rapeseeds and incubation of soil

Rapeseeds of ERM-BC367 (colza, Sigma-Aldrich) contain 99 mmol kg^-1^ total GSLs with ~61% 2-hydroxy-3-butenyl-GSL (progoitrin) and ~25% 3-butenyl GSL (gluconapin) as major components [7]. Rapeseeds (1.5 g) were extracted with 10 mL of water by homogenizing with pestle and mortar. HPLC analysis revealed that the aqueous rapeseed extracts contained 33.8 ± 12.8 μmol progoitrin and 0.6 ± 0.3 μmol gluconapin per 1.5 g seeds (mean ± SD, n = 6), other GLSs were present in traces (S1 Fig) [7,18,19].

Since the aqueous rapeseed extract also contains myrosinase activity (~17.7 ± 3.4 m*U*/seed; ~0.15 ± 0.03 *U* mg^-1^ protein; determined by HPLC), goitrin was formed after hydrolyzation and cyclization of the unstable 2-hydroxy-3-butenyl ITC. For standardization, the extracts (10 mL) were incubated with 0.15 *U* of myrosinase (from *Sinapis alba*, Sigma-Aldrich) for 30 min at room temperature to convert all GSLs into ITCs. A cell-free homogenate designated "rapeseed-extract" (RS-EX) was obtained by filtration through Miracloth. The RS-EX contained only minor amounts of progoitrin (0.9 ± 0.7 μmol) and gluconapin (0.6 ± 0.7 μmol) but 21 ± 2 μmol goitrin per 1.5 g seeds (S1 Fig). Sinapine (sinapic acid choline ester) is a bioactive, phenolic compound in rapeseed. The total sinapic acid content after alkaline hydrolysis measured by HPLC was 22.0 ± 1.5 μmol in 1.5 g seeds (S1 Fig) [19].

Prior to treatment, the frozen soil was thawed at 4°C for 24 h. RS-EX w (10 mL derived from 1.5 g seeds) was added per pot containing 300 g soil. The soil/RS-EX mixture was incubated in the dark at 21°C in glass beakers covered with glass lids and Parafilm. During incubation, freshly prepared extracts were added to the soil every third day. Control soils were incubated with the addition of water. RS-EX treatments were carried out in three biological replicates each with three separate pots.

HPLC measurements revealed that the application of the RS-EX to the soil led to the addition of ~575 nmol goitrin and ~366 nmol sinapic acid (free or conjugated) per g soil. The goitrin concentration represents the lower limit considered to be effective in plant pest control (517–1294 nmol methyl-ITC g^-1^ soil) [20]. 24 h after application, ~60% of goitrin can be retrieved, while sinapic acid is completely degraded (S1B Fig). After 48 h, ~20% of goitrin is left which is completely degraded after 5 days. In another experiment, RS-EX was added to the soil every third day during 4 weeks, and goitrin and sinapic acid measured one week later. Only traces of goitrin and no sinapic acid were detected in the soil. Therefore, goitrin remains in the soil for 2–3 days, while sinapic acid, even after repeated RS-EX addition, is degraded in less than 24 h.

### DNA extraction, PCR amplification, Illumina MiSeq sequencing, and data analysis

Total metagenomic DNA from soil was extracted and purified using the NucleoSpin Soil kit (Macherey & Nagel, Düren, Germany). To probe the microbial communities, the 16S rRNA (U3441F-U806R) and internal transcribed spacer (ITS; ITS7F-ITS4R) regions were amplified by PCR from the bacterial or fungal genomic DNA, respectively (S5 Table) (LGC Genomics, Berlin, Germany). PCR amplicons were sequenced by Illumina MiSeq V3 and processed by LGC Genomics. Raw sequence data were analyzed using the Quantitative Insights Into Microbial Ecology software (QIIME 1.9.0) [21]. Sequences were first quality filtered by discarding reads (i) with final lengths of < 100 bp, (ii) with a Phred score below 33, or (iii) with homopolymers and ambiguous base pairs exceeding 8. The resulting quality-filtered FASTA files were merged using BBMerge 35.43. Taxonomic classification was performed employing the SILVA [22] reference classification system, and sequences were clustered into operational taxonomic units (OTUs). To predict the taxonomic distribution of the underlying microbial community of the samples, the OTU-picking strategy was adopted with a 97% sequence similarity threshold using the clustersplit method. The sequences were assigned to the lowest possible taxonomic rank with NCBI BLAST+ 2.2.29 with an identity level of > 90% for bacteria and against the UNITE version 6 reference database for fungi. A phylogenetic tree was constructed using FastTree. Alpha-diversity metrics (PD whole tree and Chao1 index) were computed. Beta-diversity was measured using UniFrac [23], and PCoA was performed using the UniFrac results. Jacknifed beta-diversity was determined to measure robustness of UPGMA clusters and clusters in the PCoA plots.

### Isolation and taxonomic classification of cultivable microorganisms

Microorganisms from soil were isolated by serial dilution and plating on selective media. Suspensions were prepared by vortexing soil and water (1:1). After centrifugation (2000x *g*, 10 min), aliquots (50 μL) of 1000 and 10,000 fold diluted samples of the supernatant were inoculated on (i) LB agar plates (1% w/v trypton, 0.5% w/v yeast extract, 0.5% w/v NaCl) containing nystatin (25 μg/mL), and (ii) malt agar plates (1.5 w/v malt extract, 1% w/v bacto agar, 0.8% w/v yeast extract, 0.5% w/v glucose, 0.5% w/v fructose) containing ampicillin (100 μg/mL). Plates were incubated for several days at 22°C. Discrete bacterial and fungal colonies were selected and their purity was tested by streaking to new LB or malt agar plates. Colonies were picked and used for PCR analyses with the primers for 16S rRNA (U3441F-U806R) or ITS/IGS (ITS7F-ITS4R) for bacteria or fungi, respectively [24]. PCR products were purified using the NucleoSpin Gel and PCR Clean-up Kit (Macherey & Nagel, Düren, Germany) and sequenced. Sequences were searched against the nucleotide database at Genbank using BLASTN. Cultivable bacteria and fungi were stored at -80°C as glycerol cryostocks. Microorganisms were tested for (i) their abilities to solubilize phosphate from Ca_3_(PO_4_)_2_, (ii) their plant growth promoting activities with *Arabidopsis* seedlings, and (iii) their capacity to cope with goitrin and sinapic acid.

### Phospholipid extraction and analysis from soil samples

Phospholipids were isolated from total lipid extracts from soil samples [25,26] by solid phase extraction on silica columns [27]. After extraction, soil samples were dried for 24 h at 105°C to determine the soil moisture content and dry weight.

For phospholipid fatty acid (PLFA) analysis, fatty acids of the isolated phospholipids were converted into methyl esters [28] in the presence of internal standard (5μg tridecanoic acid, 13:0). Fatty acid methyl esters were quantified using an Agilent 7890 gas chromatograph with Supelco SP-2380 column and a flame ionization detector. Fatty acid peaks were identified using standards (Supelco 37 Component FAME Mix, Sigma) and confirmed by gas chromatography-mass spectrometry using an Agilent 7890 with a 30 m HP-5MS column. Mass spectra were compared to the National Institute of Standards and Technology (NIST) library. PLFAs (or combinations thereof) were assigned to different taxonomic groups [29] (S2 Table).

The phospholipid content and molecular species composition was determined by direct infusion mass spectrometry on an Agilent Accurate Mass quadrupole time-of-flight (Q-TOF) mass spectrometer [26,27]. A standard mix containing two molecular species each of phosphatidylinositol (PI), phosphatidylserine (PS), phosphatidylethanolamine (PE), phosphatidylcholine (PC), phosphatidylglycerol (PG) and phosphatidic acid (PA) was added. The phospholipid extract was infused into the Q-TOF mass spectrometer using the Agilent ChipCube nanospray source. Phospholipid molecular species were quantified by MS/MS experiments [30].

### Analysis of soil enzymes

Enzymes were isolated from soil samples following extraction protocols of native proteins with silica adsorption/desorption [31,32]. Soil samples (10 g) were mixed with 20 mL of buffer I (100 mM KH_2_PO_4_/K_2_HPO_4_ pH 7, 10 mM NaCl) and incubated for 1 h at 25°C. Solid material was collected by centrifugation and re-extracted with 10 mL of buffer I (containing 1 mg/mL polyvinylpyrrolidone K90). Supernatants of the two extractions were combined and concentrated tenfold by ultrafiltration (5 kDa cutoff) at 4°C.

Esterase/lipase activities were measured by incubating 50 μL of soil extracts with 200 μL of *p*-nitrophenyl butyrate (20 mM final concentration) in potassium phosphate buffer (pH 7.2) (90 min, 37°C) [33]. One unit of esterase/lipase activity corresponds to the amount of enzyme releasing 1.0 μmol min^-1^ of *p*-nitrophenol (ε = 10,400 M^−1^ cm^−1^). *In vitro* refolded lipase LipA from *Pseudomonas aeruginosa* served as control [34]. Phospholipase A activities were measured by quantification of fatty acids released from di-lauroyl-PC using the NEFA-HR-Kit (Wako Chemicals, Neuss, Germany). Soil extracts (10 μL) were incubated with 10 μL of di-lauroyl-PC (1.3 mM) in 40 mM Tris-HCl, 1% Triton X-100, pH 7.2 with for 8 h at 37°C. One unit of phospholipase A activity is defined as the amount of enzyme releasing 1.0 μmol min^-1^ of lauric acid. Phospholipase A PA2949 from *Pseudomonas aeruginosa* was used as control [35]. Protease activity was determined by degradation of fluorescein-tagged casein using the Thermo fluorescent protease assay kit (Thermo Scientific) with 25 μL soil extract. One unit of protease activity is defined as the amount of enzyme extract releasing 1.0 μmol min^-1^ of fluorescein labeled peptide. Trypsin was used as control.

### Determination of GLS and sinapic acid contents and myrosinase activity in rapeseed extracts (RS-EX)

To measure the GLS content of the aqueous RS-EX extract, 0.3 g of BCR-367R rapeseeds were homogenized with 2 mL water on ice. After centrifugation at 14000 *g* at 4°C, 600 μL of RS-EX was loaded on a diethylaminoethyl (DEAE) Sephadex A-25 column and the glucosinolates eluted after desulfatation. GSLs were quantified by HPLC relative to the internal standard glucotropaeoline [19,36]. Progoitrin, gluconapin and goitrin were identified by co-chromatography with commercial standards.

Endogenous myrosinase activity with progoitrin was determined in RS-EX extracts. The assay contained 65 μL100 mM phosphate buffer (pH 6.8), 28.5 μg protein (25 μL crude RS-EX) and 260 μM progoitrin. The reaction was terminated after 10 min of incubation at 30°C by 5 min by boiling and centrifugation to remove coagulated protein. The supernatant was analyzed for goitrin production by HPLC with external standard curves.

HPLC analysis of RS-EX revealed the presence of sinapine (sinapic acid choline ester) and lower amounts of free sinapic acid and other sinapic acid conjugates. Since sinapic acid which can be released from sinapic acid conjugates by microbial enzymes, represents the most relevant bioactive compound, the total sinapic acid content was determined after alkaline hydrolysis with 2 M NaOH [37]. After acidification, the hydolysate was extracted with ethylacetate and analyzed by HPLC. The hydrolysate contained one major compound which was identified as sinapic acid by its UV spectrum and retention time in comparison with a commercial standard (Sigma).

### Plant growth promoting activities of cultivable microorganisms

Cultivable bacteria were grown in LB medium, and fungi were grown in malt medium. The strain *Shigella flexneri* was not further considered because it is a human pathogen. The bacterial isolates of *Bacillus mycoides*, *Lysinibacillus fusiformis*, *Variovorax paradoxus*, *Enterobacter*, and the fungi *Mortierella verticillata*, *Clonostachys rosea* and *Hypocrea mucoiana* lost their viability after cryoconservation.

Phosphate solubilization by the cultivable microbial strains was analyzed on Pikovskaya solid medium prepared with 2.5 g L^-1^ Ca_3_(PO_4_)_2_ using an established dot method [38]. The plates were incubated for 7 days in the dark at 25°C. Solubilization of phosphate was estimated by determination of the solubilization index, SI = (halozone diameter–colony diameter) / colony diameter.

To directly test growth promoting properties of cultivable microbial strains, three-week-old *Arabidopsis thaliana* Col-0 seedlings of the same sizes were transferred to pots filled with well fertilized soil (9 plants per strain and 15 control plants) [39,40] and inoculated once with 10 mL of the microbial strain (OD600 = 0.05 in water). Under these conditions, phosphate solubilization is not required for plant growth. Controls were treated with 10 mL of water. The plants were grown under greenhouse conditions and watered every second day. Three weeks after inoculation, photos were taken and the rosettes harvested for FW determination.

### Incubation of the cultivable microorganisms with goitrin or sinapic acid

Cultivable microbial strains (500 μL of OD600 = 0.1) precultured in LB medium were transferred to15 mL Czapek medium containing 121 μmol goitrin at 25°C in the dark. Furthermore, cultivable microbial strains were also grown in 15 mL Czapek medium containing 72 μmol sinapic acid. Growth was recorded by measuring the OD600 at 1, 2 and 5 days after inoculation. Then, the goitrin or sinapic acid contents in the medium were measured by HPLC after 2 or 5 days.

## Results

### PLFA analysis reveals irreversible changes in microbial diversity in RS-EX treated soil

**Fig 1:**
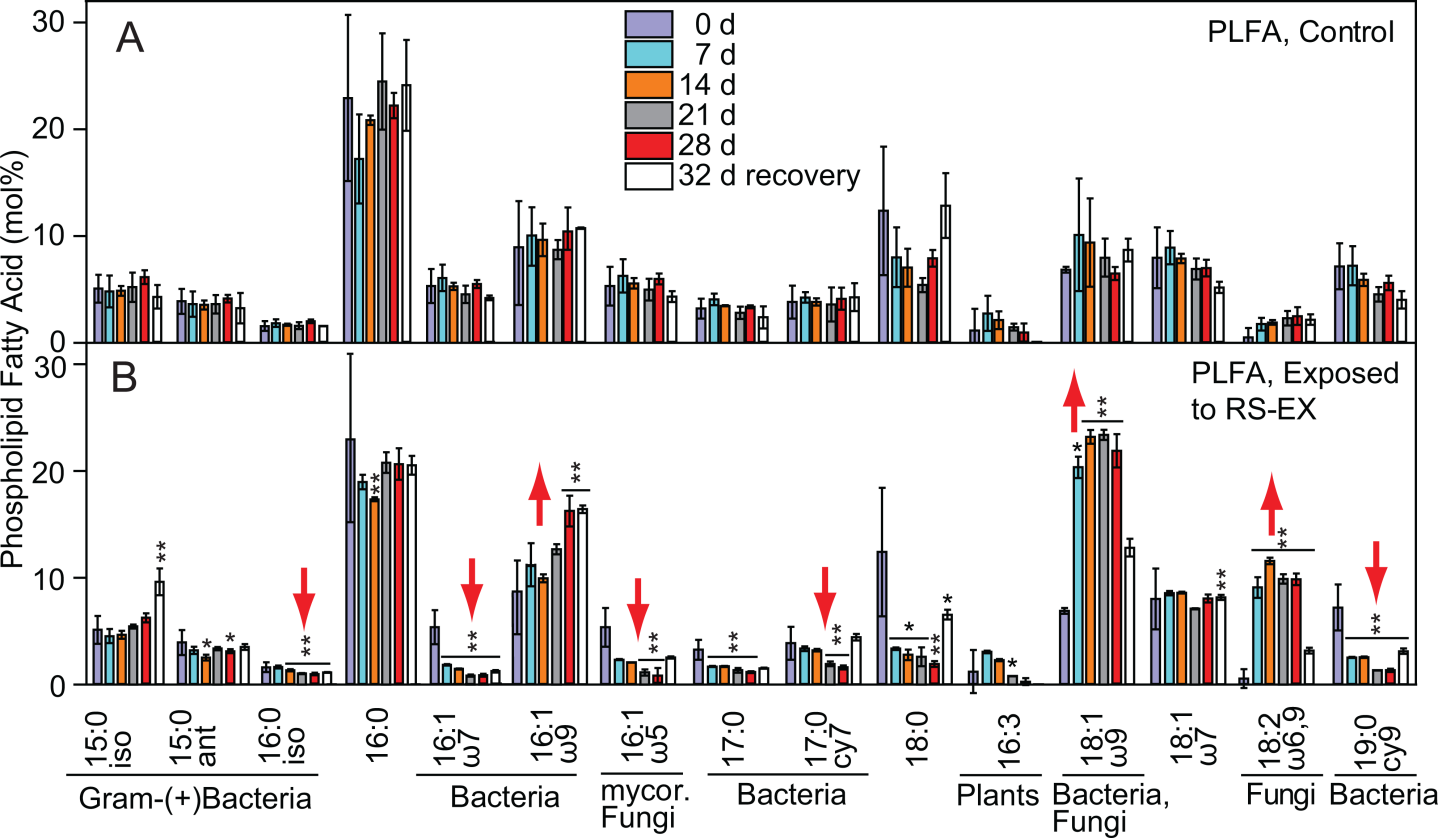
Decreased abundances of mycorrhizal fungi and Gram-negative bacteria during RS-EX treatment as determined by phospholipid fatty acid (PLFA) analyses of soil samples. PLFA analyses of A. untreated control soil samples and B. of soil during RS-EX exposure were performed by GC-MS measurements. Marker fatty acids for different taxonomic groups are listed in S2 Table. Data show means ± SD (n = 3). Significant differences between RS-EX treated and control samples are indicated by asterisks (t-test; *, p<0.05; **, p<0.005). 32 d recovery, 28 d of RS-EX treatment followed by 32 d without treatment. Arrows indicate increased or decreased amounts of bacterial or fungal PLFAs after RS-EX application.

To study the impact of a standardized rapeseeds extract (RS-EX) containing predominantly the cylic ITC goitrin, on bacterial and fungal community structures, soil without a history of glucosinolates was treated with RS-EX followed by PLFA profiling as an indicator of microbial diversity. (S1 Table; Fig 1A and 1B). The measurement of the phospholipid fatty acid (PLFA) composition was employed for the analysis of microbial diversity, because specific PLFAs are characteristic for bacterial and fungal taxa [41]. For example, methyl-branched fatty acids (15:0iso, 15:0ant, 16:0iso; for abbreviations see S2 Table) are mainly found in Gram-positive bacteria [42]. Cyclopropane fatty acids (17:0cy, 19:0cy) are indicative for bacteria exposed to stress [43]. Odd-chain (e.g. 17:0) and the monounsaturated PLFAs 16:1ω7 and 16:1ω9 are general biomarkers for bacteria. 18:1ω9 is found in bacteria and fungi, while 18:2ω6,9 serves as fungal biomarker [29,44,45], and 16:1ω5 is found in arbuscular mycorrhizal fungi [46]. Polyunsaturated fatty acids (16:3, 18:3) are markers for cyanobacteria or eukaryotic algae.

The relative amounts of many fatty acids prevalent in bacteria (16:0iso, 16:1ω7, 17:0cy, 19:0cy) decreased after exposure to RS-EX (Fig 1), whereas the amounts of other bacterial (16:1ω9) and bacterial/fungal (18:1ω9) fatty acids increased. The relative abundance of arbuscular mycorrhizal fungi as deduced from 16:1ω5 contents decreased by ~60% after 28 d of RS-EX treatment, suggesting a relative decline in mycorrhizal fungi compared with other fungi (18:1ω9 and 18:2ω6,9).

We performed a recovery experiment where the soil that treated for 28 d with RS-EX was incubated for further 32 d with the addition of water (Fig 1). Most of the bacterial PLFA biomarkers (16:0iso, 16:1ω7, 17:0, 19:0cy) and the mycorrhizal fungal marker (16:1ω5) remained at low levels, indicating that the changes were irreversible and that many microorganisms were fully extinct.

### Massive death of soil microbiota is accompanied with the increase in phosphatidic acid and hydrolytic enzyme activities

**Fig 2:**
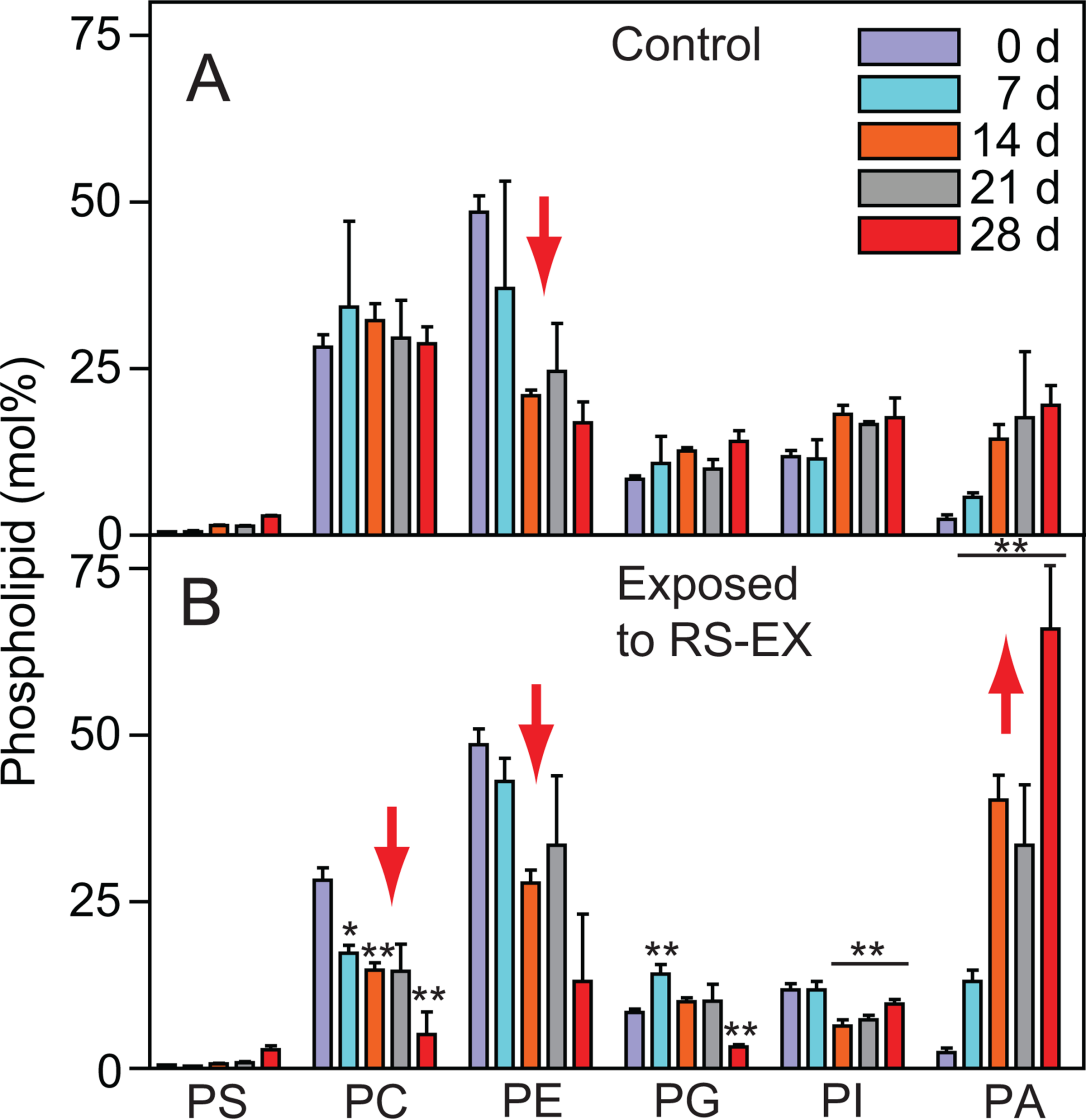
Accumulation of phosphatidic acid indicates decomposition of microbial soil organisms during RS-EX treatment. Alterations in phospholipid composition of A. control and B. RS-EX treated soils as determined by Q-TOF mass spectrometry. Bars in panels A and B represent means ± SD (n = 3). Significant differences to control are indicated by asterisks (**, p ≤ 0.05). PA, phosphatidic acid; PC, phosphatidylcholine; PE, phosphatidylethanolamine; PG, phosphatidylglycerol; PI, phosphatidylinositol. Red arrows indicate increased or decreased amounts of bacterial or fungal phospholipids after RS-EX application.

While the determination of PLFAs was used to assess global changes in soil microbial diversity, information on the abundances of microbial species cannot be collected because many fatty acids occur in more than one taxonomic group. The isolation and measurement of native phospholipids by mass spectrometry provides quantitative information about the contents and molecular species composition of the different phospholipid classes including phosphatidylcholine (PC), phosphatidylethanolamine (PE), phosphatidylglycerol (PG), phosphatidylserine (PS) and phosphatidylinositol (PI) [27]. Mass spectrometry can be employed to assess the phospholipid composition of the microorganisms in whole soil samples [26]. Thus, differences in phospholipid composition between bacteria and fungi can be recorded to disclose low-resolution changes in the soil microbial community structure during RS-EX application. Phospholipids, membrane components of all organisms, are rapidly degraded after cell death by (phospho-)lipases, yielding fatty acids, lysophospholipids, diacylglycerol and in particular phosphatidic acid (PA). Therefore, PA represents an important marker for cell death, and the amounts of the intact phospholipids can be correlated with living soil microbiota [47]. In soil samples treated with RS-EX, the relative amounts of the phospholipids except PA decreased (Fig 2). PC is mainly found in eukaryotes (fungi, nematodes, animals), but it also occurs in few bacteria, including members of Actinobacteria and Alphaproteobacteria [48,49]. The relative amount of PC stayed constant in the control soils, yet it continually decreased to less than 20% of the control at the end of the RS-EX treatment suggesting a loss of fungal biomass. PE, an abundant component in bacterial and fungal membranes decreased in control and RS-EX treated soils. Most strikingly, the content of the cell-death marker PA strongly increased during RS-EX treatment. Therefore, changes in phospholipid composition in the soil indicate a loss in the richness of bacterial and fungal species, caused by massive cell death.

**Fig 3:**
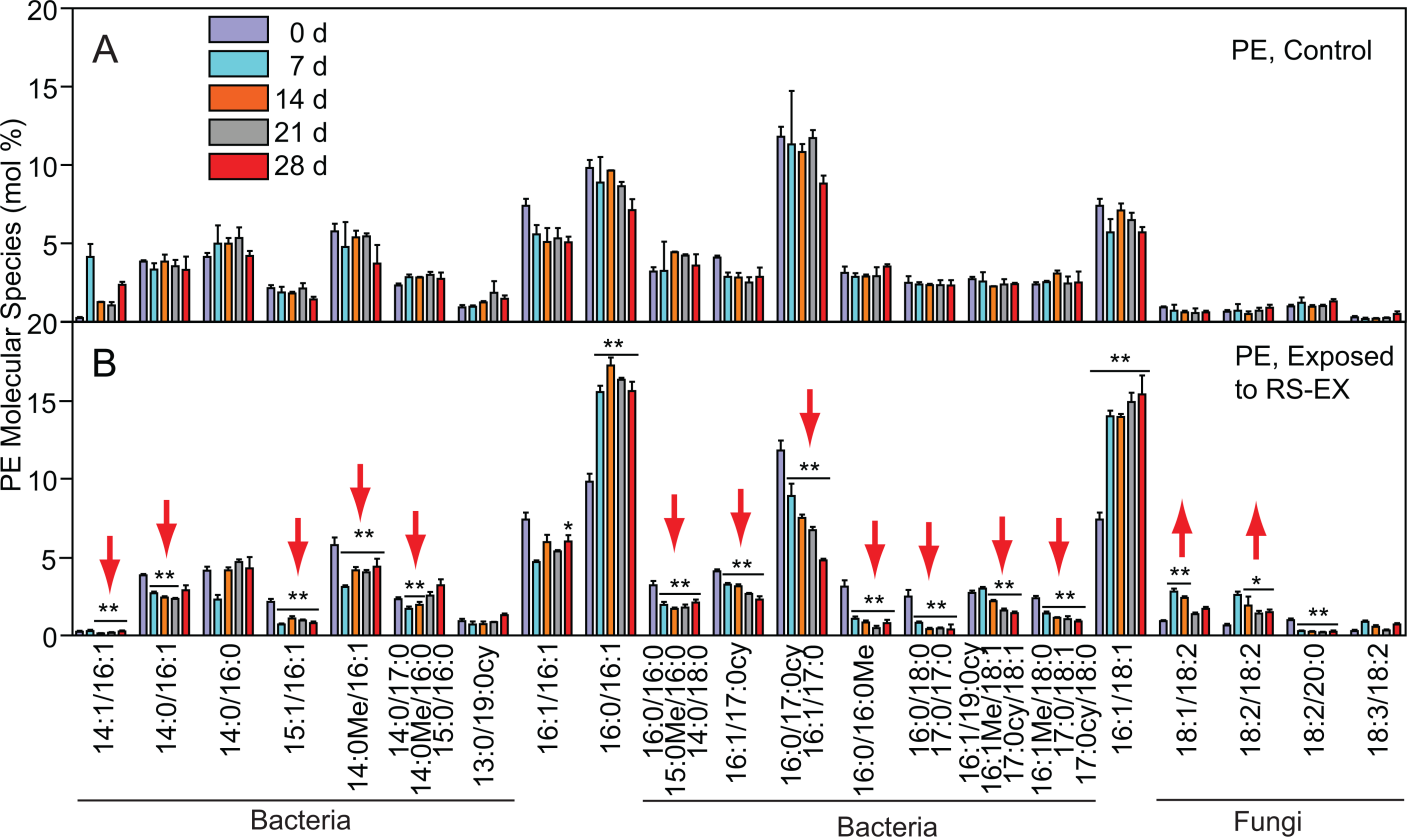
Alterations in bacterial and fungal community structures during RS-EX treatment revealed by alterations in phosphatidylethanolamine (PE) molecular species composition. Changes in PE molecular species in A. control and B. RS-EX treated soil samples were measured by Q-TOF mass spectrometry. Markers for bacteria (Bact) and fungi are indicated. Means ± SD are shown (n = 3). Asterisks indicate significant differences to control (t-test; *, p < 0.05; **, p < 0.005). Fatty acids of molecular species are indicated in S2 Table. Arrows indicate increased or decreased amounts of bacterial or fungal molecular species of PE after RS-EX application.

Because many phospholipid classes, e.g. PC and PE, can be derived from different taxa, analysis of the molecular species composition can provide more detailed information about their origin. Molecular phospholipid species containing 14:1, 15:1, 16:1, 18:1, 14:0Me, 16:0Me, 17:0cy or 19:0cy acyl groups are characteristic for bacteria, while 18:2 and 18:3 containing phospholipid molecular species are found in fungi (S2 Table) [26,50]. The relative abundances of bacterial molecular species of PE, PC, PI and PG decreased during RS-EX treatment, indicating a decline in bacterial biomass (Figs 3 and S2–S4). The increase in relative amounts of fungal phospholipids after RS-EX exposure, in particular PC containing 18:2 or 18:3, as compared to bacterial phospholipids, indicates that the fungi were less affected than bacteria. Conclusively, the results obtained from PLFA and phospholipid analysis demonstrate a strong decline in microbial biodiversity with differential losses of bacterial and fungal taxa.

**Fig 4:**
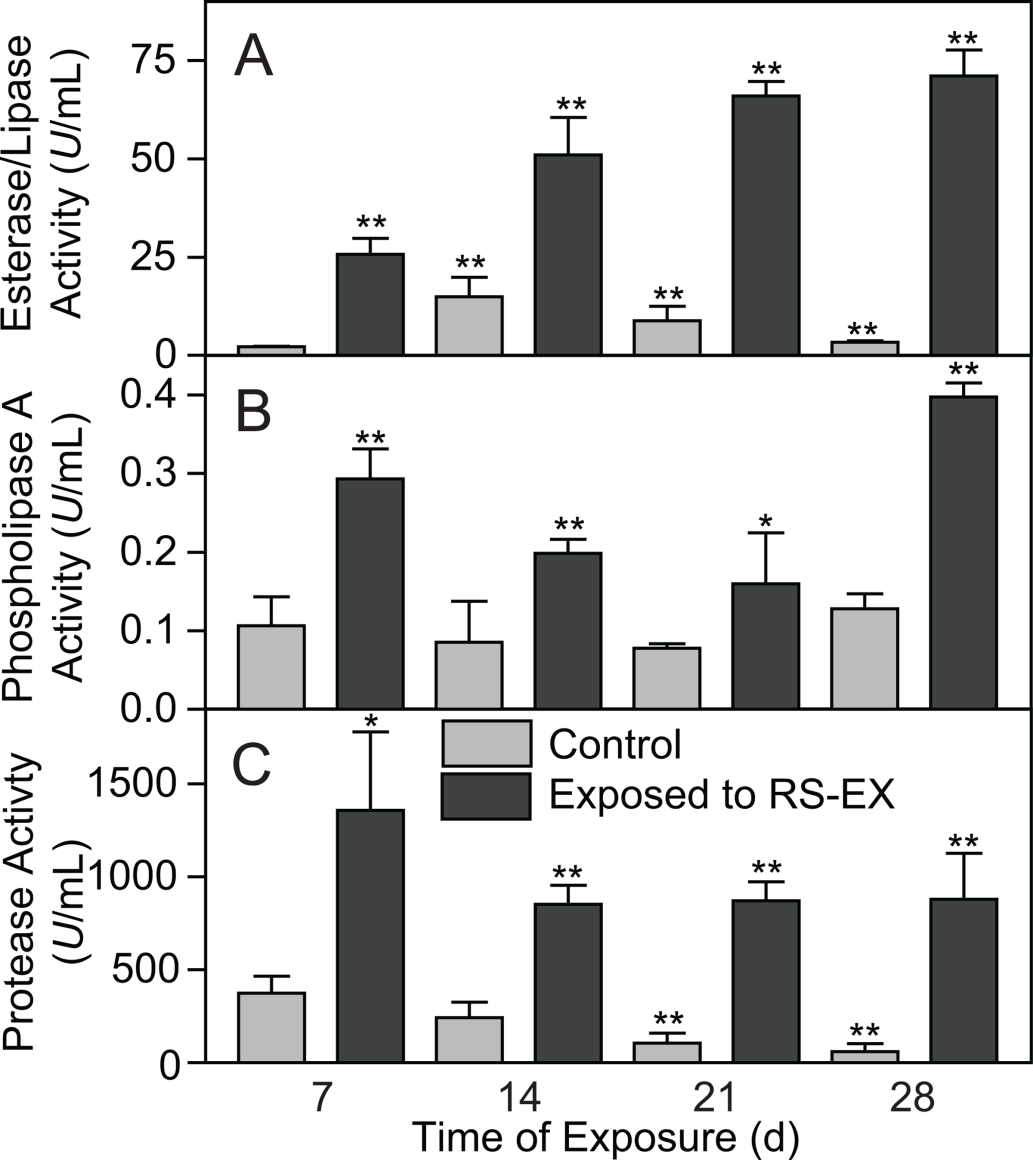
Changes in microbial enzyme activities during RS-EX treatment of soils. The activities for A. esterases/lipases, B. phospholipases A and C. proteases are shown for control (grey bars) and RS-EX treated (black bars) soil extracts. Data show mean ± SD (n = 3). Significant differences of the control or RS-EX treated measurements at the different days to the control samples at 7 d are indicated (t test; *, p<0.05; **, p<0.005).

To study the impact of RS-EX application on the metabolism of microbial organisms, three enzymatic activities ubiquitous among bacteria and fungi were quantified in soil extracts. Application of RS-EX to the soil strongly increased the activities of esterases/lipases, phospholipases A (PLA) and proteases (Fig 4). During the course of RS-EX treatment, the esterase/lipase activity was further increased, while PLA and protease activities were variable but always higher than the control. Possibly, the increase in hydrolytic activities reflects the production and secretion of enzymes involved in utilization of carbon from dying biomass and might therefore explain the decreases in phospholipid molecular species, accompanied with the massive accumulation of PA (Fig 2).

### Next generation sequencing discloses a decline in microbial diversity

DNA Sequencing was conducted at five time points (0, 7, 14, 21 and 28 d) for RS-EX treated and untreated soils. Abundances of the prokaryotic taxonomical groups for all time points are shown in S3 Table. For sake of simplicity and easier graphical presentation of highly diverse bacterial communities, the results of all time points (controls versus RS-EX treatment) were combined.

Alpha-diversity can be calculated according to different aspects of the community structure (S5 Fig). The soil microbiota revealed a total of 11,066 and 561 OTUs (97% sequence identity) for bacteria/archaea and fungi, respectively. The average number of observed OTUs deduced from rarefaction curves (S5A Fig) showed that the sequencing depth encompassed most of the phylotype richness in each sample. The fungal diversity was largely covered with 12,000 sequences per sample. For bacteria, the rarefaction curve did not reach an asymptote even with 2,000 or 8,000 sequences per sample, indicating high bacterial diversity in the sample which could be elucidated by a larger sequencing effort.

The Chao1 index, an average alpha-diversity parameter for richness [51], was reduced, indicating a decline in species richness after RS-EX treatment compared to non-treated controls for bacteria (p = 0.003) and fungi (p = 0.007) (S5 Fig). Similarly, the phylogenetic diversity (PD) whole tree indices differed significantly between RS-EX treated and control soils for bacteria (p = 0.003) or fungi (p = 0.002) (S5B Fig). The decline in the number of taxa in RS-EX treated samples is in agreement with the results obtained by PLFA and phospholipid analyses, indicating massive cell death of the soil microbiota and a decrease in diversity. Principal coordinate analysis (PCoA) of beta-diversity (based on weighted and unweighted UniFrac distances) of soil treated with RS-EX and untreated soil was performed to estimate the phylogenetic differences between the samples. The analyses based on the relative abundance of OTUs revealed that the communities in RS-EX treated and control soils were phylogenetically distinct (S6 Fig). Finally, Unweighted Pair Group Method with Arithmetic Mean (UPGMA) hierarchical clustering of the weighted UniFrac distances demonstrated a grouping pattern similar to that obtained by principal coordinate analysis for bacteria and fungi.

### Reshaping of bacterial and archaeal community diversities after RS-EX exposure

**Fig 5:**
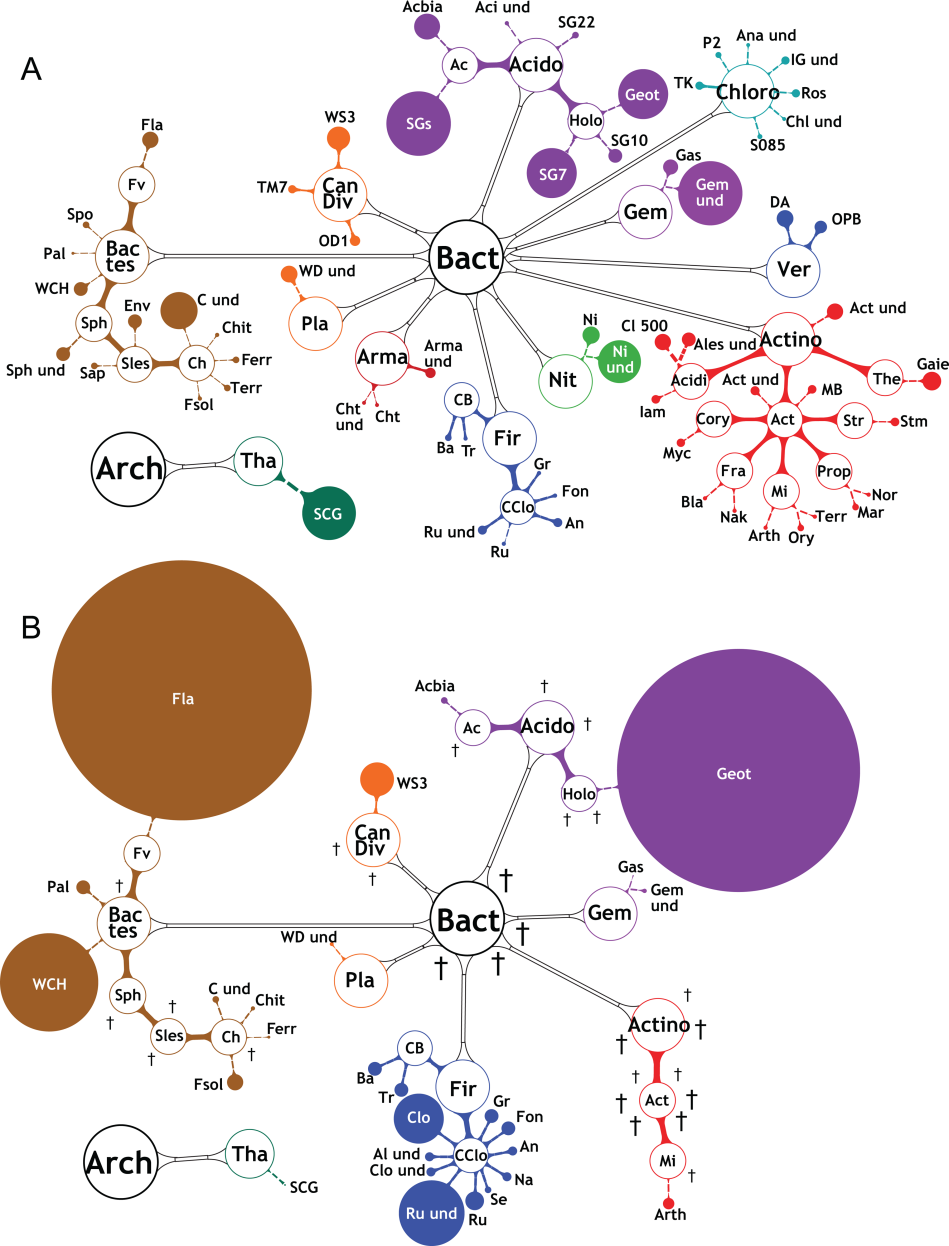
Changes in archaeal and bacterial diversity during RS-EX treatment. The bacterial and archaeal diversity of A. control (average of 0–28 d), and B. RS-EX treated soil (average of 0–28 d) is shown. Relative OTU abundances derived from the numbers of 16S RNA reads are indicated by the sizes of filled circles. Open circles indicate higher-order taxa (circle sizes not in scale). Extinction of OTUs is depicted by crosses. Arch, Archaea; Bact, Bacteria; Acido, Acidobacteria; Actino, Actinobacteria; Arma, Armatimonadetes; Can Div, Candidate Division; Chlo, Chloroflexi; Bactes, Bacteriodetes; Fir, Firmicutes; Gem, Gemmatimonadetes; Nit, Nitrospirae; Pla, Planctomycetes; Ver, Verrucomicrobia; Tha, Thaumarchaeota. Other taxa are listed in S3 Table.

**Fig 6:**
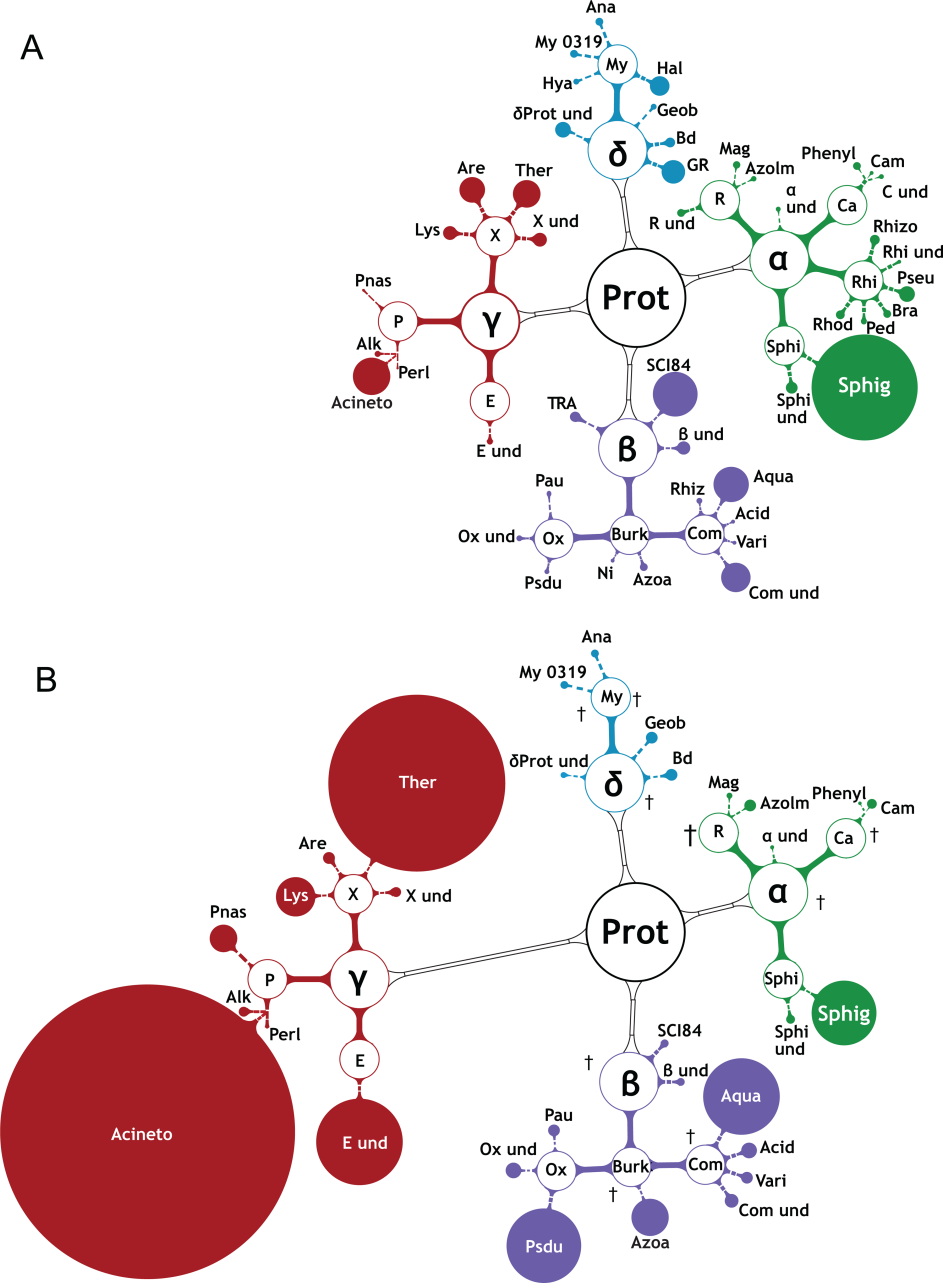
Changes in abundance of soil α, β, γ, δ Proteobacteria (Prot). The figure shows the relative abundances of A. control samples (averages of 0 d—28 d) and B. of RS-EX treated soils (averages of 0 d -28 d). The sizes of the filled circles indicate abundances of soil bacterial OTUs derived from 16S rRNA gene sequencing. Open circles depict higher-order taxa (circle sizes not in scale). Crosses indicate extinction of OTUs. The abundances of *Acinetobacter* (Acineto) and *Thermomonas* (Ther) dramatically increased after RS-EX treatment. Taxa are listed in S3 Table.

RS-EX treatment of soils resulted in strong changes in biodiversity at the bacterial phylum level with Proteobacteria (α, β, γ, δ) and Acidobacteria being most abundant in the untreated and RS-EX treated soils (S7 Fig). Proteobacteria proliferated occupying about 60% of the soil bacterial community after 28 d of RS-EX treatment. The relative abundances of Firmicutes and Bacteriodetes strongly increased, while the abundance of Acidobacteria remained slightly decreased during RS-EX application. All other phyla strongly decreased or were extinct (S7 Fig). Untreated control soils showed high bacterial species diversity with low numbers of individuals (Figs 5 and 6 and S3 Table). Upon RS-EX treatment, these bacteria were among the first which decreased in favor of few taxa that prevailed. Many subgroups of the *Acidobacteria* (Acido), including Holophagaceae (Holo) and Acidobacteriales (Ac) were afflicted (Fig 5). The *Actinobacteria* (Actino), including members of the Iamiaceae (Iam), Micrococcaceae (Mi) and Streptomycetaceae (Str) were strongly affected or extinguished. Members of the Bacteroidetes, (Bactes) (e.g. Sphingobacteriales (Sles), the Chloroflexi (Chloro), Gemmatimonadetes (Gem), and Nitrospirae (Nit)) were extinct after RS-EX application (Fig 5). Similar results were obtained with members of the Verrucomicrobia (Ver) and of Candidate Division (Can Div OD1, WS3). The members of the Candidate Division TM7 [52] increased transiently, but were finally extinct. Numerous Alphaproteobacteria (α; e.g. Rhizobiales, Rhi; Rhodospirillales, R), Betaproteobacteria (β; e.g. Nitrosomonadaceae, Ni) and Deltaproteobacteria (δ) were also extinguished after RS-EX application (Fig 6).

On the other hand, the abundances of certain species of *Flavobacterium (Fla)* and B1-32 unclassified (WCH) (both Bacteriodetes, Bates), and *Geothrix* (Geot, of Acidobacteria, Acido) dramatically increased after RS-EX exposure (Fig 5). Although of low abundance, species of the *Bacillus* (Ba), *Clostridium* (Clo) and unclassified Ruminococcaceae (Ru und) (of Firmicutes, Fir) became transiently enriched in RS-EX treated soil (Fig 5 and S3 Table). Betaproteobacteria species of the *Pseudoduganella* (Psdu), *Aquabacterium* (Aqua), *Azospira* (Azoa) and *Variovorax* (Vari) strongly proliferated after RS-EX application (Fig 6). Members of the Gammaproteobacteria (γ) became the predominant bacterial phylum with strong increases of *Acinetobacter* (Acineto), Enterobacteriales (E und) *Pseudomonas* (Pnas), *Thermomonas* (Ther) and *Lysinibacillus* (Lys). Interestingly, a number of genera proliferating after RS-EX treatment contain species previously shown to harbor growth promoting properties, i.e. *Flavobacterium*, *Bacillus*, *Clostridium*, *Variovorax*, *Acinetobacter*, *Enterobacterium* and *Lysinibacillus* (Table 1).

**Table 1:** Taxonomic classification of cultivable bacteria and fungi.

Taxon	Query Cover [%]	Identity [%]	PGP	Comment	Reference
**Bacteria**					
**Bacteriodetes**					
*Chitinophaga niastensis*	99	97	PGP	degrading polysaccharides	[53]
**Actinobacteria**					
*Mycobacterium fortuitum (7)*	100	88	PGP	human pathogen, in biochar	[54]
**Firmicutes**					
*Bacillus* *aryabhattai (34)*	99	99	PGP		[55]
*Bacillus* *cereus (59)*	99	99	-	antibiotic producing	
*Bacillus* *megaterium (21*, *48)*	100	99	PGP		[56]
*Bacillus* *methylotrophicus (58)*	100	99	PGP	methylotrophic	[57]
*Bacillus* *mycoides*	98	99	PGP		[58]
*Lysinibacillus fusiformis*	100	98	PGP		[59]
*Lysinibacillus xylanilyticus (56)*	100	98	PGP	degrading polysaccharides	[60]
*Paenibacillus polymyxa (51)*	99	99	PGP	pathogen antogonist	[61]
*Solibacillus silvestris*	100	98	PGP	quorum quenching	[62]
**Alphaproteobacteria**					
*Aminobacter aminovorans (49)*	99	99	-	methylotrophic, auxin producing	[63]
**Betaproteobacteria**					
*Variovorax* *paradoxus*	100	99	PGP	quorum quenching	[64]
**Gammaproteobacteria**					
*Acinetobacter* *kookii*	100	99	-	-	
*Enterobacter* *sp*.	97	94	PGP		[65]
*Pseudomonas* *frederikbergensis*	99	99	-	-	
*Pseudomonas syringae*	100	100	-	plant pathogen	[66]
*Shigella flexneri*	100	99	-	human pathogen	
**Fungi**					
**Zygomycota**					
*Mortierella verticillata*	99	99	PGP	degrading polysaccharides	[67]
**Ascomycota**					
*Clonostachys rosea*	100	96	PGP	pathogen antagonist	
*Hypocrea muroiana*	99	96	PGP	produces antibiotics	[68]
*Papulaspora sepedonioides (12)*	13	94	-	-	
*Pseudogymnoascus roseus*	98	93	-	pathogen stimulant	
*Trichoderma viride (47)*	99	91	PGP	degrading GSLs	[69]

Bacteria and fungi cultivated from RS-EX soil were identified by comparison of 16S rRNA and IGS/ITS sequences, respectively, with nucleotide sequences at Genbank. Underlined genera were also identified by sequencing of soil samples after RS-EX treatment. Numbers in brackets depict strain isolates. PGP, plant growth promoting.

### Prolonged RS-EX exposure affects the soil fungal diversity

**Fig 7:**
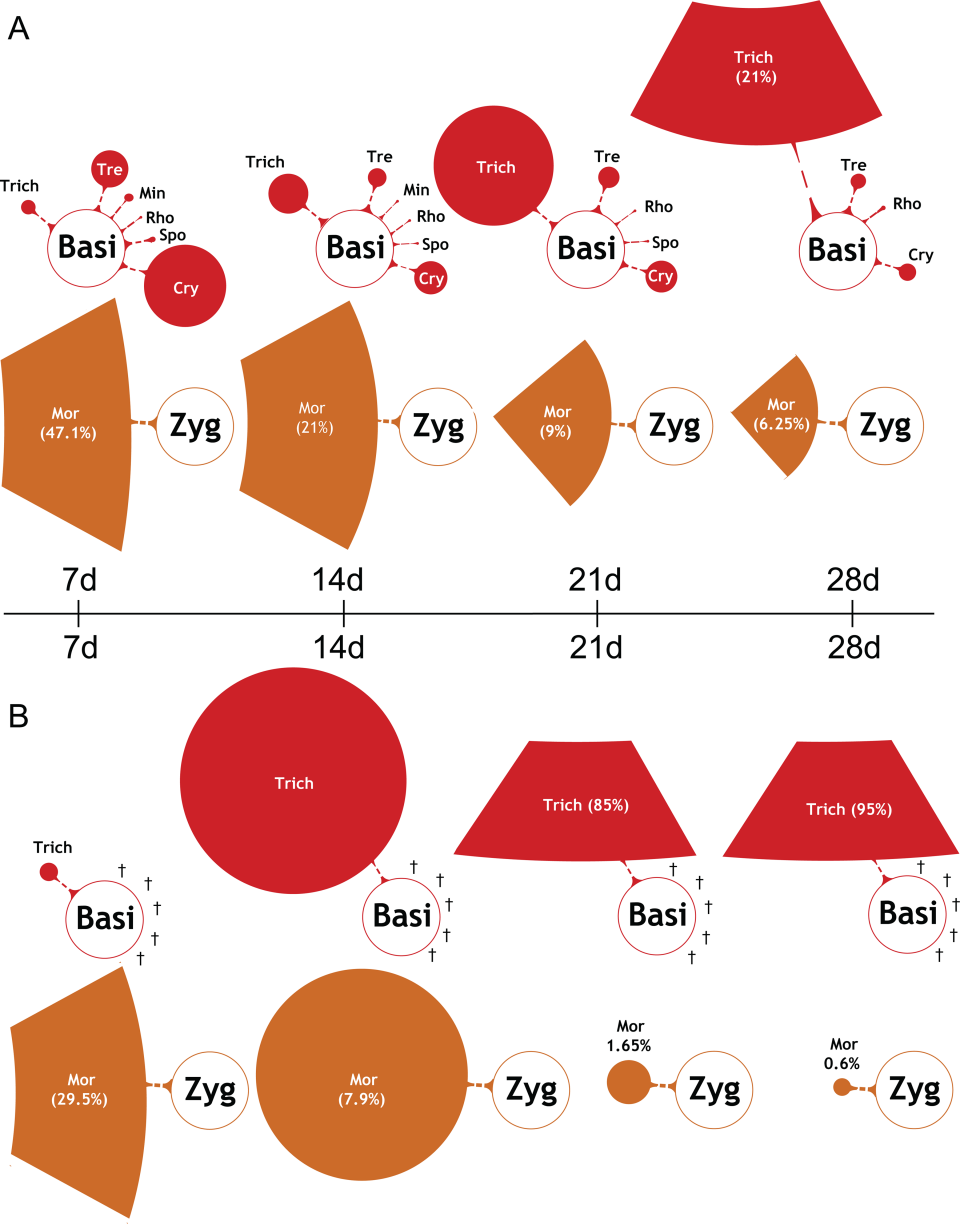
Changes in the community composition of basidiomycota and zygomycota. Basidiomycota (Basi) and Zygomycota (Zyg) OTU abundances derived from internal transcribed spacer (ITS) region sequencing in A. control soil samples and B. during RS-EX exposure (7, 14, 21, 28 d). Filled circle sizes indicate average OTU abundances. Highly abundant taxa are depicted by circle sections, and abundances given in %. Open circles indicate higher-order taxa (circle sizes not in scale). Extinction of OTUs is depicted by crosses. Trich, *Trichosporon*; Mor, *Mortierella*. Other taxa are listed in S4 Table.

**Fig 8:**
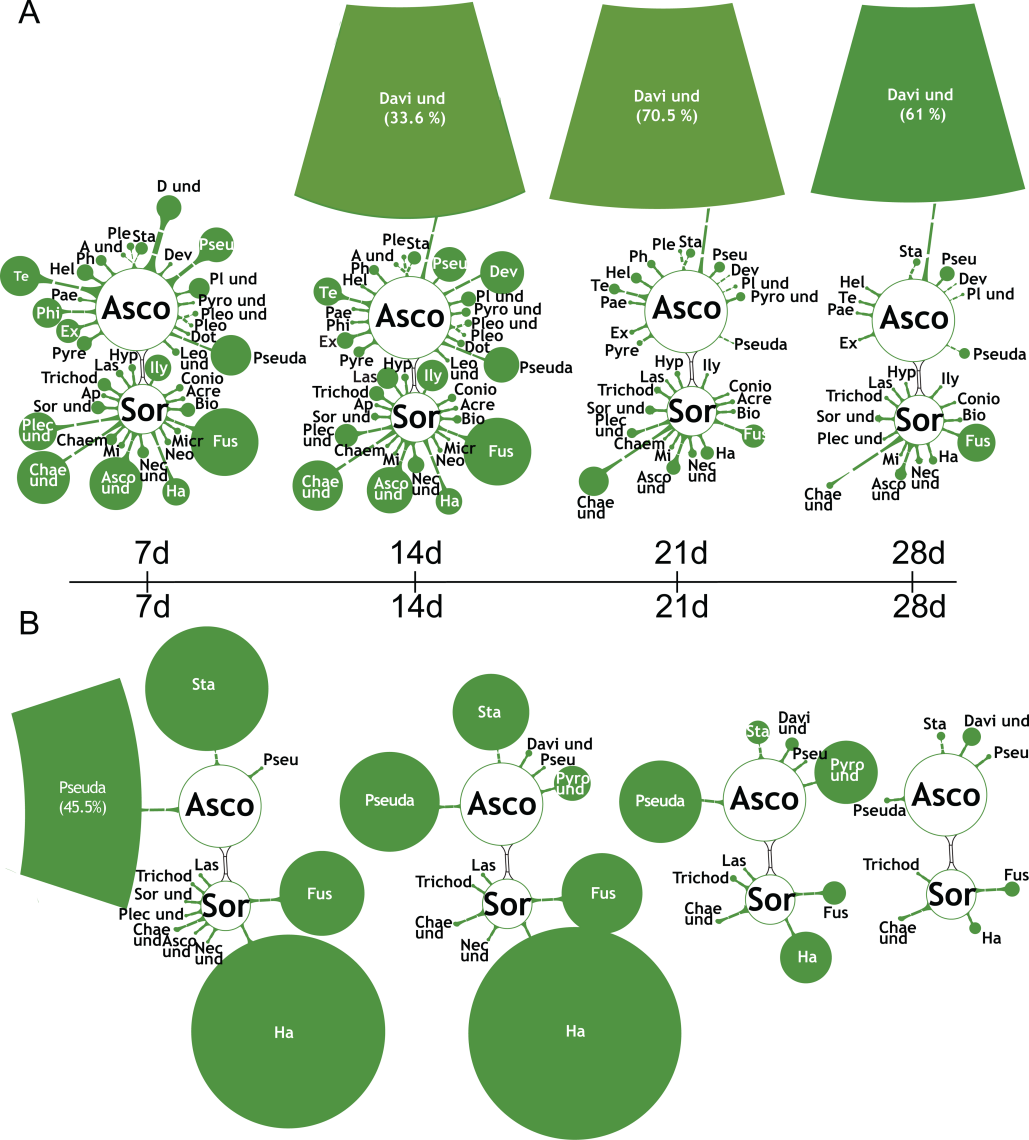
Breakdown of ascomycota diversity during RS-EX exposure. Changes in Ascomycota (Asco) OTU abundances derived from internal transcribed spacer (ITS) region sequencing in soil samples of A. controls and B. during RS-EX exposure (7, 14, 21, 28 d). Average OTU abundances are indicated by the sizes of filled circles. Highly abundant taxa are depicted by circle sections, and abundances given in %. Open circles indicate higher-order taxa (circle sizes not in scale). Extinction of OTUs is depicted by crosses. Davi und, Davidiellaceae unclassified; Ha, Haematonectria; Pseuda, Pseudaleuria; Sor, Sordariomycetes; Sta, Staphylotrichum. Other taxa are listed in S4 Table.

The fungal biodiversity massively declined during RS-EX treatment. Ascomycota and Zygomycota were the most abundant phyla in the untreated and RS-EX treated soils at 7 d (S8 Fig). While Ascomycota fungi further proliferated in the untreated soil, Ascomycota and Zygomycota were almost completely extinct after RS-EX application, and Basidiomycota prevailed. Saprophytic *Mortierella* (Mor) species of the Zygomycota (Zyg), important for organic matter turnover, were highly abundant in control soil (47.1% of fungal DNA at 7 d), but they were reduced to 6.3% at 28 d (Fig 7A). Seven-day treatment with RS-EX decreased the relative abundance of *Mortierella* to 29.5%, and after 28 days, it was almost extinct. At the same time, *Trichosporon* (Trich) (Basidiomycota, Basi) increased in treated samples, representing ~95% of the fungal DNA at 28 d (Fig 7B). *Trichosporon* also increased in the control soils, but not earlier than after 21 d, and to a much lesser extent (21% at 28 d) (Fig 7A). This genus contains species such as *T*. *asahii* with growth promoting effects (Table 1). Members of the Ascomycota (Asco) were the most diverse fungal group in the control soils (Fig 8A). They were almost completely extinguished by RS-EX after 28 d (Fig 8B). Species of the *Pseudaleuria*, *Staphylotrichum*, *Fusarium* and *Haematonectria* represented the major portion of the total fungal DNA during the first 14 days of RS-EX treatment, but prolonged input of RS-EX drastically reduced the number of these taxa. In conclusion, 28 d of RS-EX application caused the disappearance of almost all Ascomycota and Zygomycota (i.e. *Mortierella*), with one genus, *Trichosporon* of the Basidiomycota, dominating the fungal microbiota.

### Isolation of RS-EX-tolerant bacteria and fungi revealed the presence of plant growth promoting microorganisms

Cultivable microorganisms were isolated from the RS-EX treated soil and taxonomically classified after PCR amplification and sequencing (Tables 1, S6 and S7). Most of the isolated bacteria belong to the Firmicutes, Proteobacteria, and Bacteriodetes. Interestingly, many bacterial species were previously shown to harbor plant growth promoting properties, like the Gram-positive *Bacillus cereus*, *Bacillus methylotrophicus* and *Bacillus mycoides* [70,71], as well *as Enterobacter hormaechei* (Gammaproteobacteria) [72]. While many fungi were extinct after RS-EX treatment, some fungal species of the Ascomycota survived, including *Trichoderma viride*. *Trichoderma* species can degrade GSLs from rapeseed meals [73], and *Trichoderma viride* belongs to the plant growth promoting *Trichoderma* species, some of which producing auxins or antibiotics against pathogenic fungi [69,74] (Table 1).

Plant growth promoting properties of microorganisms are often strain specific. Most noticeable, taxonomic levels of surviving and cultivable microorganisms were of low abundance or even undetectable in soil samples by DNA sequencing (S3 and S4 Tables), except for the Enterobacteriaceae which accumulated and the genus *Bacillus* with a low transient enrichment in RS-EX treated soil (S7 Table). The genera *Trichoderma* and *Papulaspora* (unclassified Sordariomycetes) are low abundant in the RS-EX treated samples. To experimentally confirm the growth promoting activities, cultivable microorganisms were first analyzed for their ability to solubilize phosphate from insoluble tribasic calcium phosphate (Ca_3_(PO_4_)_2_), a property characteristic for many plant growth promoting microorganisms [38] (S9 Fig). The phosphate solubilization (indicated by cell growth) was highest with *Aminobacter aminovorans* (strain 49), *Paenibacillus polymyxa* (51) and *Bacillus methylotrophicus* (58), followed by the two strains of *Bacillus megaterium* (21, 48), *Bacillus aryabhattai* (34) *and Trichoderma viride* (47). *Bacillus cereus* (59), *Lysinibacillus xylanilyticus* (56) and *Papulaspora sepedonioides* (12) grew without distinct halozone. *Mycobacterium fortuitum* (7) did not grow on (Ca_3_(PO_4_)_2_). The results indicate that eight out of eleven cultivable strains are able to solubilize phosphate from insoluble Ca_3_(PO_4_)_2_ which represents a potent plant growth promoting property.

**Fig 9:**
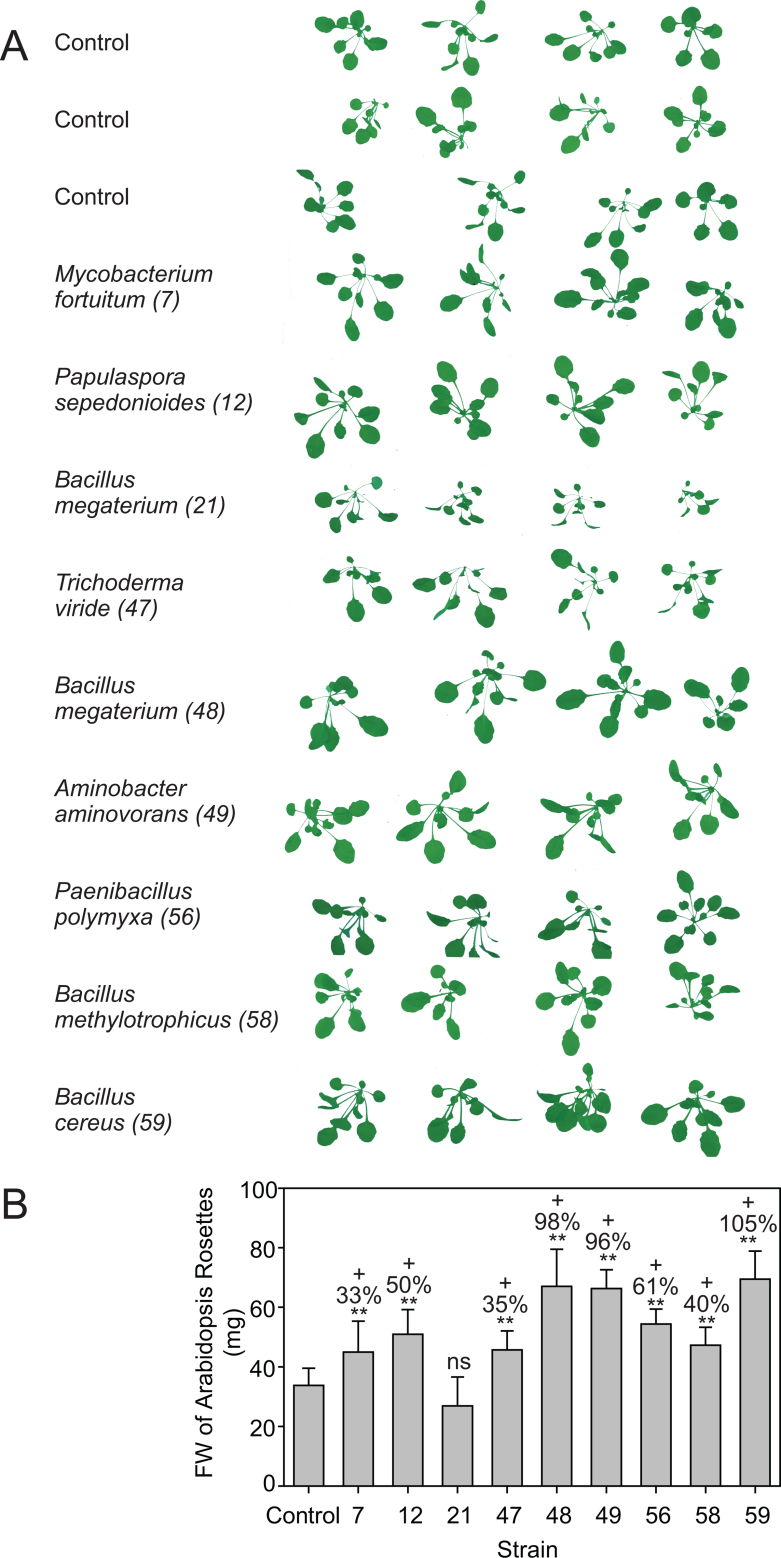
Increase in *Arabidopsis* growth after inoculation with cultivable bacteria from RS-EX treated soils. A. Three-week-old *Arabidopsis* Col-0 seedlings growing on soil were inoculated with 10 mL of a bacteria suspension (OD600 = 0.05) and photos of the rosettes taken 3 weeks later. Control plants were inoculated with water. Numbers in brackets depict strain number. B. Fresh weights (FW) of *Arabidopsis* rosettes. *Bacillus megaterium* (48), *Aminobacter aminovorans* (49) and *Bacillus cereus* (59) exert the strongest growth promoting effect. Bars (means ± SD; n = 4). Numbers above bars indicate % change to control. Asterisks indicate significant differences to the control (**, p<0.01).

While it was not the purpose of our study to examine the process of root colonization or to characterize colonization genes [75,76], the plant growth promoting activities of the strains were recorded directly. *Arabidopsis thaliana* Col-0 plants were inoculated with the cultivable microorganisms, and after three weeks, photos were taken followed by determination of the rosette FW. Except for *Bacillus megaterium* (21), all tested microorganisms caused an increase in plant biomass accumulation (Fig 9). *Bacillus megaterium* (strain 48), *Aminobacter aminovorans* (49) and *Bacillus cereus* (59) most strongly increased aboveground biomass production, followed by *Papulaspora sepedonioides* (12), *Lysinibacillus xylanilyticus* (56), *Bacillus methylotrophicus* (58), *Mycobacterium fortuitum* (7) and *Trichoderma viride*,(47). Therefore, the cultivable microbial strains directly promote plant growth, presumably by mechanisms different from phosphate solubilization.

Finally, the capacity of the cultivable microorganisms to degrade goitrin or sinapic acid when cultured in Czapek medium, and the impact of goitrin or sinapic acid on growth were tested. Microbial strains were incubated with goitrin, and the goitrin content in the medium was determined after 2 or 5 d of growth. Most of the microorganisms degraded large amounts of goitrin within 5 days (S10A Fig). Strains 21 and 34 showed low goitrin degradation capacity. The growth of the microorganisms was hardly influenced by goitrin (S10B Fig), except for strain 58 which showed poor growth during the first days. All strains rapidly metabolized sinapic acid in 2 days, except *Bacillus methylotrophicus* (58), which needed 5 days (S10A Fig). Growth in the presence of sinapic acid was not altered (not shown). Thus, most cultivable species are able to degrade goitrin and all of them metabolized sinapic acid thus in part explaining why these two secondary metabolites had only minor effects on the growth of cultivable microorganisms.

## Discussion

Using high throughput lipidomic and enzymatic approaches combined with next generation sequencing, we present a holistic study on the effects of RS-EX application to the soil. Our studies unravel a dramatic loss of microbial diversity, in agreement with previous experiments using genomic sequencing methods only [13,14]. In addition, we show that specific microbial taxa harboring plant growth promoting properties survived (Table 1, Fig 9). The combination of complementary strategies for the analysis of live (lipidomics, enzymology) or total (DNA sequencing) biomass is essential to analyze and understand microbial community dynamics. The conclusion is proven e.g. by the fact that mycorrhizal fungi OTUs could not be detected by next generation sequencing, but by lipidomics. The latter method together with enzymology also provides evidence for microbial metabolic activity or dying microorganisms. Here, the isolation of cultivable bacterial and fungal species after RS-EX treatment, which included a number of plant growth promoting microorganisms, represents a starting point for understanding interspecies relationships in soil in response to RS-EX, and in particular to the rapeseed derived cyclic ITC, goitrin.

In addition to GSL, rapeseeds contain further secondary metabolites, in particular phenolics. Sinapic acid is the major phenolic with amounts comparable to those of progoitrin (~30 μmol in 1.5 g RS-EX, S1 Fig). We considered the possibility that sinapic acid might also affect microbial growth, in addition to rapeseed-derived progoitrin. Sinapic acid is one of the most common phenolics in the plant kingdom. Therefore, the soil microbiota is constantly exposed to sinapic acid derivatives. Moreover, sinapic acid is biodegradable by many bacteria and fungi [77–80] (S10 Fig). Therefore, it is unlikely that sinapic acid is responsible for *Brassica*-specific biocidal properties on the soil microbiota. However, it is possible that sinapic acid, in combination with goitrin, shows synergistic or additive growth inhibiting effects. At present, it is unclear why goitrin-tolerant microorganisms did not proliferate much stronger in the soil during prolonged RS-EX treatment. Possibly, these microorganisms need to be associated with living *Brassica* plants, or they were compromised by the release of unknown antibiotic compounds from the accompanying bacteria and fungi that were stressed and dying. PLFA analysis has widely been used for the characterization of microbial organisms in soils in response to land management and environmental cues [81]. While easy to perform, it is difficult to distinguish different species within complex microbial mixtures due to the presence of many specific fatty acids in the different taxa.

The identification of 16:1ω5 provided evidence for the relative decrease in abundance during RS-EX treatment of mycorrhizal fungi (e.g. Glomeromycota) in agreement with the fact that Brassicaceae including rapeseed cannot be colonized by mycorrhizal fungi. While no sequence hits for mycorrhizal fungi were found, this result indicates that fatty acid analysis of soil samples can unravel the presence of microbial organisms not accessible by DNA sequencing. Possibly, the harsh procedure of lipid extraction allows the capture of organisms, including spores, which may be inaccessible by aqueous extractions employed for DNA sequencing.

The characterization and quantification of phospholipid molecular species from soil by mass spectrometry represents a distinct approach unraveling further details on the effects of RS-EX on the soil microbiota. The reduction in the amounts of different phospholipids is in accordance with the decrease in biodiversity. The accumulation of PA clearly demonstrated that RS-EX treatment caused the death of microorganisms, rather than just inhibiting their growth. Furthermore, the increase in hydrolytic enzyme activities in the soil suggested that some microorganisms metabolized dead biomass. The utilization of dying biomass can contribute to the dynamic occurrence of some microorganisms [82]. Consequently, the measurements of phospholipids and enzyme activities provide important results for the understanding of soil ecophysiology that could not have been provided by DNA sequence analysis alone.

Bacterial species that grow on or even metabolize GSLs (e.g. sinigrin) have previously been isolated [83]. Several of the bacteria described there were also isolated and cultivated after RS-EX treatment in the present study, including *Pseudomonas syringae* (Gammaproteobacteria) and members of the Enterobacteriales, *Clostridium*, *Bacillus* and *Arthrobacter* (Actinobacteria) [84]. The ubiquitous Gram-positive Rhizobacterium *Bacillus cereus* has been previously isolated after rapeseed extract treatment [85]. However, other presumably GSL-tolerant bacteria (*Agrobacterium*, *Micrococcus*, *Sinorhizobium*) were not retrieved in the present study, apparently because they were absent from the original soil (Figs 5 and 6).

*M*. *verticillata* (Zygomycota) was the most abundant fungal species in control and RS-EX soils at 7 d of treatment. The decrease in abundance of *M*. *verticillata* in control and treated soils presumably reflects the exhaustion of decaying material during the incubation period, as many *Mortierella* species are saprophytes. *Haematonectria* represents the teleomorphic form of *Fusarium* [86]. The result that the abundance of *Fusarium* was decreased during prolonged RS-EX treatment is in agreement with the finding that biofumigation with Brassicaceae plant material represents an efficient strategy for the control of pathogenic *Fusarium* species [87]. Incubation of the control soil resulted in a strong proliferation of the Davidiellaceae family, with *Davidiella*, the teleomorphic form of *Cladosporium*, representing one of the most common environmental fungi [88]. *Trichosporon* species proliferated in control soils, amounting to 21% after 28 d of incubation. However, the proportion of *Trichosporon* accumulated to even 95% of fungal biomass after RS-EX treatment, thus, representing most of the remaining fungal biodiversity. *Trichosporon* includes yeast species growing on soil as well as human pathogens, e.g. *Trichosporon cutaneum* [89]. In agreement with the strong proliferation during RS-EX treatment, *Trichosporon* was previously shown to be GSL resistant [85]. Although yet poorly investigated, fungal strategies for GSL detoxification encompass the increase of myrosinase activity, or the conjugation of ITCs with glutathione catalyzed by glutathione S-transferases [90]. In addition to other systems including thioredoxin/thioredoxin reductases, the latter strategy is also known for bacteria [91]. These results provide the basis for the genetic analysis of the strategies employed by microorganisms to cope with toxic GSL.

Plants shape their rhizosphere microbiome by exudation of secondary metabolites which serve as chemical attraction for specific microorganisms [92–95]. We hypothesize that *Brassica* plants establish beneficial microbial consortia adapted to GSLs and ITCs. With respect to the given habitat, there might be a more constant and a more variable portion of microbial species synergistically optimizing plant growth, as a result of co-evolutionary processes [96–98]. Habitat specialists, identified by indicator species analysis at OTU level, emphasize the rapidity by which plants shape their associated microbial consortia. The considerable number of plant growth promoting microorganisms identified by sequencing and after cultivation (Table 1) indicates that even in bulk soil previously not used for *Brassica* growth, ITC/goitrin tolerant beneficial microorganisms are present, albeit at low abundance. However, as we did not test for the presence of plant growth promoting strains in the control soil, it is difficult to claim that such strains were enriched during ITC application. These findings are in agreement with previous results [98] that showed a higher heterogeneity of the microbiome with a higher suppression capacity of pathogens in soil of organic farming systems in comparison to conventional soils. Therefore, the history of the soil employed for testing the effects of secondary metabolites on the soil microbiota is of critical importance.

In the future, functional studies are required to decipher the relevance of these bacteria and fungi for soil health. The loss of the microbial biodiversity represents a risk for ecosystem processes, climate and plant growth. In longer term, it lowers the evolutionary capacity for adaption of the soil microbiome to changing environmental conditions [99] resulting in the loss of soil fertility and increasing land degradation.

## Supporting information

S1 Fig HPLC chromatograms and contents of the major secondary metabolites in rapeseed. A. Glucosinolates isolated from an aqueous rapeseed extract (RS-EX) (Fang et al., 2012) without myrosinase treatment. The insets show the UV spectra for progoitrin and goitrin. B. Myrosinase-treated aqueous RS-EX used for soil treatments contains mainly sinapin and goitrin; progoitrin and gluconapin are present in traces. Detection with diode array detector (λ = 230 nm). C. An aqueous RS-EX was prepared from 1.5 g rapeseeds and glucosinolates/ITCs measured directly, or after myrosinase treatment, by HPLC. Total sinapic acid was measured after alkaline hydrolysis of conjugated sinapic acid. Bars indicate mean ± SD (n = 6). D. RS-EX was added to soil in a ratio of 1.5 g seed extract to 300 g of soil. This application was equivalent to 675 μmol goitrin and 366 μm sinapic acid per g soil. Soil samples were taken after 24 h, 48 h and 5 days and used for goitrin and sinapic acid measurements by HPLC. While goitrin was slowly degraded during ~2 days, sinapic acid was below detection limit already after 24 h.
(EPS)

S2 Fig Changes in phosphatidylcholine (PC) molecular species composition after RS-EX exposure. Changes in PC molecular species in A. control soil and B. after RS-EX treatment were recorded by Q-TOF mass spectrometry. Data show mean ± SD (n = 3). Significant differences to the control samples are indicated (t test; *, p<0.05; **, p<0.005). The acyl groups of molecular species are given in S4 Table. Arrows indicate increased or decreased amounts of bacterial or fungal molecular species of PC after RS-EX application.
(EPS)

S3 Fig Analyses of phosphatidylinositol (PI) molecular species composition after RS-EX exposure. Changes in PI molecular species in A. control soil and B. after RS-EX treatment. Data were obtained by Q-TOF mass spectrometry and show mean ± SD (n = 3). Significant differences to the control samples are indicated (t test; *, p<0.05; **, p<0.005). The acyl groups of molecular species are given in S4 Table. Arrows indicate increased or decreased amounts of bacterial or fungal molecular species of PI after RS-EX application.
(EPS)

S4 Fig Analyses of phosphatidylglycerol (PG) molecular species after RS-EX exposure. Changes in PG molecular species in A. control soil and B. after RS-EX treatment. Data were obtained by Q-TOF mass spectrometry and show mean ± SD (n = 3). Significant differences to the control samples are indicated (t test; *, p<0.05; **, p<0.005). The acyl groups of molecular species are given in S4 Table. Arrows indicate increased or decreased amounts of bacterial or fungal molecular species of PG after RS-EX application.
(EPS)

S5 Fig Alpha-diversity indices of metagenomics analyses in RS-EX treated and control soils. A. Rarefactions for the observed bacterial and fungal numbers of OTUs were calculated and rarefaction curves were generated with QIIME 1.9.0. Mean ± SD (n = 9) (sampling time points for each treatment were combined). Exposure to RS- reduces the number of bacterial and fungal OTUs in the high range of sequences per sample. B. Box plots of bacterial (left) and fungal (right) alpha-diversity indices (Chao1 index; phylogenetic diversity/PD whole tree index) for the 16S rRNA gene and 28S rRNA (ITS) sequences, respectively. The boxes display median (in red) and percentiles excluding outliers outside lower and upper extremes. Dashed error lines show SD (n = 9, time points combined).
(EPS)

S6 Fig Principle coordinate analysis (PCoA) plot showing differences in the phylogenetic diversities of the soil microbiota after RS-EX exposure. The phylogenetic diversity of the soil microbiota was assessed using UniFrac. Weighted A. and unweighted B. UniFrac Jackknifed PCoA plots of changes in microbial community composition after RS-EX treatment. Control soil (C, red), RS-EX treated soil (G, blue), not incubated (C0, orange). The numbers indicate the days of incubation. The contributions of the three major principal coordinates (PCoA1, PCoA2, PCoA3) to the diversity of the sample sets are indicated in percent.
(EPS)

S7 Fig Changes in abundances of major bacterial and archaeal phyla in the soil after RS-EX treatment. The bars show average relative abundances of phyla derived from 16S rRNA gene sequencing A. in the control soil and B. after exposure to RS-EX. On phylum levels, the influence of RS-EX is restricted to the relative increase of sequence reads for Firmicutes, Proteobacteria and Bacteroidetes, the decrease of Chloroflexi, and the intermediate decrease of Acidobacteria. The days of exposure are indicated. Total relative abundance was below 100% as OTUs with relative abundances < 1% were omitted from the calculation.
(EPS)

S8 Fig Changes in the abundances of fungal phyla in the soil after RS-EX exposure. The bars show average relative abundances of fungal phyla derived from internal transcribed spacer (ITS) region sequencing A. in control soil and B. after exposure to RS-EX (for 7 d to 28 d). On phylum level, RS-EX application leads to a strong relative reduction of sequence reads for Ascomycota, accompanied with an increase in Basidiomycota, while the abundance of Zygomycota declines in untreated and RS-EX treated soils.
(EPS)

S9 Fig Growth of microbial strains isolated from RS-EX treated soil on plates containing Ca3(PO4)2 as sole phosphate source. The bacteria were inoculated on solid Pikovskaya plates containing 2.5 g L-1 Ca3(PO4)2. After 7 days, cell growth and halo zones were observed indicating the capacity to solubilize phosphate. The numbers in the center of the plastes indicate the solubilization index, SI = (halozone diameter–colony diameter) / colony diameter. The numbers in brackets depict strain numbers. *Bacillus cereus* (59) grew well but without visible halo zone. Similarly, *Lysinibacillus xylanilyticus* (56) and *Papulaspora sepedonioides* (12) grew well without distinct halo zone. *Mycobacterium fortitum* (7) was unable to grow on Pikovskaya medium.
(EPS)

S10 Fig Growth of the RS-EX tolerant, cultivable strains in the presence of goitrin or sinapic acid. Bacteria isolated and cultured from RS-EX treated soil were grown in the presence of 121 μmol goitrin or 72 μmol sinapic acid per 15 mL of culture. A. Amounts of Goitrin and sinapic acid were measured by HPLC in the cultures after 2 and 5 days. While goitrin was only slowly metabolized, sinapic acid was rapidly degraded, except for strain 58. B. Growth (recorded by measuring optical density at 600 nm, OD600) of bacteria in medium (control) or in the presence of goitrin. Growth of most bacteria was not affected by goitrin, expect strain 58 which showed reduced growth. Strain 51 transiently produced a colored product after 1 day in thepresence of goitrin which explains the high OD600 at this time point.
(EPS)

S1 Table Parameters of soil used for RS-EX treatment experiments. The soil was obtained from Wiesengut (Siegaue, Hennef, Germany). Data from analysis by Raiffeisen Laborservice (Ormont, Germany).
(DOCX)

S2 Table Fatty acids derived from PLFA and phospholipid molecular species analysis of soil samples. (DOCX)

S3 Table Prokaryotic taxonomical groups detected in soil samples. (DOCX)

S4 Table Fungal taxonomical groups detected in soil samples. (DOCX)

S5 Table Primers used for taxonomic classification of cultivable microorganisms. (DOCX)

S6 Table Sequences of 16S RNA and ITS amplicons from isolated bacteria and fungi, respectively. (DOCX)

S7 Table Abundance of cultivable microorganisms. Abundances of the microorganisms on genera level as detected by DNA sequencing are presented for the strains analyzed for their PGP properties.
(DOCX)
