# Supplementary material for: Disruption of microbial community composition and identification of plant growth promoting microorganisms after exposure of soil to rapeseed-derived glucosinolates
Source: PLoS One. 2018 Jul 3;13(7):e0200160. doi: 10.1371/journal.pone.0200160 (PMC6029813; doi:10.1371/journal.pone.0200160)
Supplement: S1 Table — The soil was obtained from Wiesengut (Siegaue, Hennef, Germany). Data from analysis by Raiffeisen Laborservice (Ormont, Germany). (DOCX) [file pone.0200160.s011.docx]

## Supporting Information

**S1 Table. Parameters of soil used for RS-EX treatment experiments.**

The soil was obtained from Wiesengut (Siegaue, Hennef, Germany).

Data from analysis by Raiffeisen Laborservice (Ormont, Germany).

| *Soil Parameter* | *Value* |
| --- | --- |
| Humus | 1.4 % |
| C/N ratio | 9:1 |
| pH | 6.1 |
| Carbonate content | 0.92 % |
| Nutrient | Content (mg 100 g^-1^ soil) |
| Total N | 106 |
| KCl | 27 |
| Phosphate | 11 |
| Potassium | 8 |
| Magnesium | 9 |
| Boron | 0.016 |
| Copper | 0.72 |
| Manganese | 36.09 |
| Iron | 20.64 |
| Zink | 1.69 |
