# Supplementary material for: Disruption of microbial community composition and identification of plant growth promoting microorganisms after exposure of soil to rapeseed-derived glucosinolates
Source: PLoS One. 2018 Jul 3;13(7):e0200160. doi: 10.1371/journal.pone.0200160 (PMC6029813; doi:10.1371/journal.pone.0200160)
Supplement: S2 Table — (DOCX) [file pone.0200160.s012.docx]

**S2 Table.** **Fatty acids derived from PLFA and phospholipid molecular species analysis of soil samples.**

| *Abbre-viation* | *Fatty Acid* | *Formula* | *Mass* | *Characteristic taxonomic group* | *Reference* |
| --- | --- | --- | --- | --- | --- |
| 13:0 | Tridecanoic Acid | C_13_H_26_O_2_ | 214.1933 |  |  |
| 14:0 | Myristic Acid | C_14_H_28_O_2_ | 228.2089 |  |  |
| 14:1 | Tetradecenoic Acid | C_14_H_26_O_2_ | 226.1933 |  |  |
| 15:0 | Pentadecanoic Acid | C_15_H_30_O_2_ | 242.2246 |  |  |
| 14:0Me | Methyl-Myristic Acid | C_15_H_30_O_2_ | 242.2246 | Gram (+) bacteria | [1] |
| 15:0iso | 13-Methyl-Myristic Acid | C_15_H_30_O_2_ | 242.2246 | Gram (+) bacteria | [2–5] |
| 15:0ant | 12-Methyl-Myristic Acid | C_15_H_30_O_2_ | 242.2246 | Gram (+) bacteria | [2–5] |
| 15:1 | Pentadecenoic Acid | C_15_H_28_O_2_ | 240.2089 |  |  |
| 16:0 | Palmitic Acid | C_16_H_32_O_2_ | 256.2402 |  |  |
| 16:0iso | 14-Methyl-Pentadecanoic Acid | C_16_H_32_O_2_ | 256.2402 | Gram (+) bacteria | [1–5] |
| 15:0Me | Methyl-Pentadecanoic Acid | C_16_H_32_O_2_ | 256.2402 | Gram (+) bacteria | [2–5] |
| 16:1 | Hexadecenoic Acid | C_16_H_30_O_2_ | 254.2246 |  |  |
| 16:1ω5 | Palmitvaccenic Acid | C_16_H_30_O_2_ | 254.2246 | Arbuscular mycorrhizal fungi | [6,7] |
| 16:1ω7 | Palmitoleic Acid | C_16_H_30_O_2_ | 254.2246 | Bacteria | [2] |
| 16:1ω9 | Hypogeic Acid | C_16_H_30_O_2_ | 254.2246 | Bacteria, Acinetobacter | [2,5,8,9] |
| 16:2 | Hexadecadienoic Acid | C_16_H_28_O_2_ | 252.2089 |  |  |
| 16:3 | Hexadecatrienoic Acid | C_16_H_26_O_2_ | 250.1933 | Plants, Algae | [10] |
| 17:0 | Margaric Acid | C_17_H_34_O_2_ | 270.2559 | Bacteria | [5] |
| 16:0Me | Methyl-Palmitic Acid | C_17_H_34_O_2_ | 270.2559 | Gram (+) bacteria | [1–5] |
| 16:1Me | Methyl-Hexadecenoic Acid | C_17_H_32_O_2_ | 268.2402 |  |  |
| 17:0cy | Cyclopropane-Palmitic Acid | C_17_H_32_O_2_ | 268.2402 | Bacteria, e.g. Acinetobacter | [1,2,4,5,11] |
| 18:0 | Stearic Acid | C_18_H_36_O_2_ | 284.2715 |  |  |
| 18:1 | Octadecenoic Acid | C_18_H_34_O_2_ | 282.2559 |  |  |
| 18:1ω9 | Oleic Acid | C_18_H_34_O_2_ | 282.2559 | Fungi, Bacteria, e.g. Acinetobacter | [1,2,6,8,9] |
| 18:1ω7 | Vaccenic Acid | C_18_H_34_O_2_ | 282.2559 |  |  |
| 18:2 | Octadecadienoic Acid | C_18_H_32_O_2_ | 280.2402 |  |  |
| 18:2ω6,9 | ω6,9-Octadecadienoic Acid | C_18_H_32_O_2_ | 280.2402 | Fungi | [2,4,6] |
| 18:3 | Octadecatrienoic Acid | C_18_H_30_O_2_ | 278.2246 | Plants, Algae | [10] |
| 19:0 | Nonadecylic Acid | C_19_H_38_O_2_ | 298.2872 |  |  |
| 18:0Me | Methyl-Stearic Acid | C_19_H_38_O_2_ | 298.2872 |  |  |
| 19:0cy | Cyclopropane-Stearic Acid | C_19_H_36_O_2_ | 296.2715 | Bacteria | [11,12] |
| 20:0 | Arachidic Acid | C_20_H_40_O_2_ | 312.3028 |  |  |
| 20:1ω9 | Gondoic Acid | C_20_H_38_O_2_ | 310.2872 | Fungi | [6] |
| 20:1ω7 | Paullinic Acid | C_20_H_38_O_2_ | 310.2872 |  |  |
| 22:0 | Behenic acid | C_22_H_44_O_2_ | 340.3341 |  |  |
| 22:1 | Docosenoic Acid | C_22_H_42_O_2_ | 338.3184 |  |  |
| 24:0 | Lignoceric acid | C_24_H_48_O_2_ | 368.3654 |  |  |

References

1. Zelles L, Palojärvi A, Kandeler E, von Lützow M, Winter K, Bai QY. Changes in soil microbial properties and phospholipid fatty acid fractions after chloroform fumigation. Soil Biol Biochem. 1997;29(9-10):1325–36. doi: 10.1016/S0038-0717(97)00062-X.

2. Zak DR, Ringelberg DB, Pregitzer KS, Randlett DL, White DC, Curtis PS. Soil microbial communities beneath *Populus grandidentata* grown under elevated atmospheric CO_2_. Ecolog Appl. 1996;6(1):257–62. doi: 10.2307/2269568.

3. Kaneda T. Iso- and anteiso-fatty acids in bacteria: Biosynthesis, function, and taxonomic significance. Microbiol Rev. 1991;55(2).

4. Bardgett RD, Hobbs PJ, Frostegård Å. Changes in soil fungal: Bacterial biomass ratios following reductions in the intensity of management of an upland grassland. Biol Fert Soils. 1996;22(3):261–4. doi: 10.1007/BF00382522.

5. Frostegård Å, Bååth E. The use of phospholipid fatty acid analysis to estimate bacterial and fungal biomass in soil. Biol Fert Soils. 1996;22(1-2):59–65. doi: 10.1007/BF00384433.

6. Madan R, Pankhurst C, Hawke B, Smith S. Use of fatty acids for identification of AM fungi and estimation of the biomass of AM spores in soil. Soil Biol Biochem. 2002;34(1):125–8. doi: 10.1016/S0038-0717(01)00151-1.

7. Olsson PA, Bååth E, Jakobsen I, Söderström B. The use of phospholipid and neutral lipid fatty acids to estimate biomass of arbuscular mycorrhizal fungi in soil. Mycol Res. 1995;99(5):623–9. doi: 10.1016/S0953-7562(09)80723-5.

8. Nishida I, Murata N. Chilling sensitivity in plants and cyanobacteria: The crucial contribution of membrane lipids. Annu Rev Plant Physiol Plant Mol Biol. 1996;47:541–68.

9. Yang C, Guo ZB, Du ZM, Yang HY, Bi YJ, Wang GQ, et al. Cellular fatty acids as chemical markers for differentiation of *Acinetobacter baumannii* and *Acinetobacter calcoaceticus*. Biomed Environ Sci. 2012;25(6):711–7. doi: 10.3967/0895-3988.2012.06.014.

10. James AT, Nichols BW. Lipids of photosynthetic systems. Nature. 1966;210(5034):372–5. doi: 10.1038/210372a0.

11. Alvarez-Ordóñez A, Fernández A, Lopez M, Arenas R, Bernardo A. Modifications in membrane fatty acid composition of *Salmonella typhimurium* in response to growth conditions and their effect on heat resistance. Int J Food Microbiol. 2008;123(3):212–9. doi: 10.1016/j.ijfoodmicro.2008.01.015.

12. Frostegård Å, Tunlid A, Bååth E. Use and misuse of PLFA measurements in soils. Soil Biol Biochem. 2011;43(8):1621–5. doi: 10.1016/j.soilbio.2010.11.021.
