# Supplementary material for: Disruption of microbial community composition and identification of plant growth promoting microorganisms after exposure of soil to rapeseed-derived glucosinolates
Source: PLoS One. 2018 Jul 3;13(7):e0200160. doi: 10.1371/journal.pone.0200160 (PMC6029813; doi:10.1371/journal.pone.0200160)
Supplement: S3 Table — (DOCX) [file pone.0200160.s013.docx]

**S3 Table. Prokaryotic taxonomical groups detected in soil samples.**

|  |  | *Abundance control %* | | | | | *Abundance RS-EX %* | | | | |
| --- | --- | --- | --- | --- | --- | --- | --- | --- | --- | --- | --- |
| *Abbreviation* | *Taxonomic Group* | *7d* | *14d* | *21d* | *28d* | *mean of all time points* | *7d* | *14d* | *21d* | *28d* | *mean of all time points* |
| **Bact** | **Bacteria (Domain)** |  |  |  |  |  |  |  |  |  |  |
| **Prot** | **Proteobacteria (Phylum)** |  |  |  |  |  |  |  |  |  |  |
| **α** | **Alphaproteobacteria** |  |  |  |  |  |  |  |  |  |  |
| Ca | Caulobacterales |  |  |  |  |  |  |  |  |  |  |
| R | Rhodospirillales |  |  |  |  |  |  |  |  |  |  |
| Rhi | Rhizobiales |  |  |  |  |  |  |  |  |  |  |
| Sphi | Sphingomonadales |  |  |  |  |  |  |  |  |  |  |
| α und | Alphaproteobacteria unclas. | 0.3 | 0.2 | 0.1 | 0.13 | 0.15 | 0.1 | 0.2 | 0.05 | 0.06 | 0.1 |
| Azolm | [Azospirillum](javascript:gg('Azospirillum');) | 0 | 0 | 0.1 | 0.4 | 0.2 | 0 | 0 | 0.5 | 1.2 | 0.5 |
| Bra | Bradyrhizobium | 0.7 | 0.25 | 0.25 | 0.2 | 0.3 | 0.25 | 0.2 | 0.15 | 0.1 | 0.1 |
| Cam | Caulobacter | 0 | 0 | 0.05 | 0.23 | 0.1 | 0.4 | 0.3 | 0.35 | 0.3 | 0.4 |
| Mag | [Magnetospirillum](javascript:gg('Magnetospirillum');) | 0 | 0 | 0 | 1.4 | 0.3 | 0 | 0.05 | 0.1 | 0.73 | 0.2 |
| Ped | Pedomicrobium | 0.25 | 0.15 | 0.2 | 0.13 | 0.2 | 0 | 0 | 0 | 0 | 0 |
| Phenyl | Phenyllobacterium | 0.2 | 0.3 | 0.35 | 0.4 | 0.3 | 0.15 | 0.15 | 0 | 0 | 0.08 |
| Pseu | Pseudolabrys | 1.05 | 0.75 | 0.55 | 0.5 | 0.7 | 0.2 | 0.1 | 0.05 | 0.1 | 0.1 |
| Rhodles und | Rhodospirillales unclas. | 0.2 | 0.35 | 0.2 | 0.23 | 0.3 | 0 | 0 | 0 | 0 | 0 |
| Rhi und | Rhizobiales unclas. | 0.35 | 0.1 | 0.15 | 0.1 | 0.1 | 0.1 | 0.1 | 0.05 | 0 | 0 |
| Rhizo | Rhizomicrobium | 0.35 | 0.55 | 0.45 | 0.37 | 0.4 | 0.1 | 0.05 | 0 | 0 | 0 |
| Rhod | Rhodoplanes | 0.35 | 0.3 | 0.35 | 0.37 | 0.35 | 0.05 | 0 | 0 | 0 | 0 |
| Sphi und | Sphingomonadales unclas. | 0.75 | 0.65 | 0.3 | 0.3 | 0.5 | 0.05 | 0.45 | 0.4 | 0.1 | 0.35 |
| Sphig | Sphingomonas | 7.45 | 7.9 | 3.9 | 3.3 | 5.3 | 8.3 | 7.0 | 1.7 | 1.1 | 3.2 |
| C und | Caulobacteraceae unclas. | 0.1 | 0.2 | 0.2 | 0.13 | 0.15 | 0.1 | 0 | 0 | 0 | 0 |
|  |  |  |  |  |  |  |  |  |  |  |  |
| **β** | **Betaproteobacteria** |  |  |  |  |  |  |  |  |  |  |
| Burk | Burkholderiales |  |  |  |  |  |  |  |  |  |  |
| Com | Comamonadaceae |  |  |  |  |  |  |  |  |  |  |
| Ox | Oxalobacteraceae |  |  |  |  |  |  |  |  |  |  |
| β und | Betaproteobacteria unclas. |  |  |  |  | 0.6 |  |  |  |  | 0.3 |
| Acid | Acidovorax | 0 | 0 | 0.2 | 0.2 | 0.1 | 0.05 | 1.95 | 0.8 | 0.7 | 0.7 |
| Aqua | Aquabacterium | 0 | 0.05 | 1.55 | 4 | 1.7 | 0.05 | 0 | 5.1 | 7.9 | 3.8 |
| Azoa | Azospira | 0 | 0 | 0.5 | 0.15 | 0.3 | 0 | 2.85 | 5.05 | 0.93 | 1.8 |
| Com und | Comamonadaceae unclas. | 1.0 | 1.3 | 1.7 | 1.3 | 1.4 | 0.85 | 0.5 | 0.3 | 0.4 | 0.6 |
| Ni | Nitrosomonadaceae unclas. | 0.35 | 0.5 | 0.65 | 0.43 | 0.1 | 0.1 | 0 | 0 | 0 | 0 |
| Ox und | Oxalobacteraceae unclas. | 0.2 | 0.15 | 0.3 | 0.23 | 0.2 | 2.65 | 0.55 | 0 | 0 | 0.7 |
| Pau | Paucimonas | 0 | 0 | 0.05 | 0.13 | 0.1 | 0.35 | 1.25 | 0.4 | 0.23 | 0.5 |
| Psdu | Pseudoduganella | 0.05 | 0.1 | 0.2 | 0.2 | 0.15 | 14.4 | 2.35 | 0.05 | 0 | 3.7 |
| Rhiz | Rhizobacter | 0.2 | 0.25 | 0.2 | 0.13 | 0.2 | 0.15 | 0.1 | 0.05 | 0 | 0 |
| SCI84 | SC-I-84 unclas. | 2.2 | 2.35 | 2.4 | 2.0 | 2.2 | 0.45 | 0.15 | 0.2 | 0.13 | 0.3 |
| TRA | TRA3-20 unclas. | 0.15 | 0.25 | 0.15 | 0.17 | 0.2 | 0 | 0 | 0 | 0 | 0 |
| Vari | Variovorax | 0.1 | 0.1 | 0.1 | 0.07 | 0.1 | 0.9 | 0.5 | 0.2 | 0.17 | 0.5 |
|  |  |  |  |  |  |  |  |  |  |  |  |
| **γ** | **Gammaproteobacteria** |  |  |  |  |  |  |  |  |  |  |
| E | Enterobacteriales |  |  |  |  |  |  |  |  |  |  |
| P | Pseudomonadales |  |  |  |  |  |  |  |  |  |  |
| X | Xanthomonadales |  |  |  |  |  |  |  |  |  |  |
| Acineto | Acinetobacter | 0 | 0 | 4.2 | 2.9 | 1.9 | 0.03 | 0.5 | 24.3 | 27.8 | 15 |
| Alk | Alkanindiges | 0 | 0.05 | 0.55 | 0.17 | 0.3 | 0.45 | 1.7 | 0.2 | 0.06 | 0.5 |
| Are | Arenimonas | 1.4 | 1.9 | 1.7 | 1.37 | 1.3 | 1.2 | 1.0 | 0.25 | 0.17 | 0.6 |
| E und | Enterobacteriaceae unclas. | 0 | 0.05 | 0.45 | 0.4 | 0.3 | 0.15 | 13.3 | 5.2 | 1.07 | 4.4 |
| Lys | Lysobacter | 0.6 | 0.6 | 0.55 | 1 | 0.7 | 1.95 | 1.75 | 0.6 | 3.3 | 2 |
| Perl | Perlucidibaca | 0 | 0 | 0.1 | 0.2 | 0.1 | 0.4 | 0.3 | 0.1 | 0.1 | 0.2 |
| Pnas | Pseudomonas | 0.05 | 0 | 0.1 | 0.2 | 0.1 | 3.9 | 1.65 | 0.25 | 0.23 | 1.4 |
| Ther | Thermomonas | 0.55 | 0.6 | 1.5 | 2.7 | 1.4 | 2.15 | 19.5 | 9.25 | 5.1 | 9 |
| X und | Xanthomonadales unclas. | 0.6 | 0.75 | 0.7 | 0.68 | 0.7 | 0.75 | 1 | 0.25 | 0.17 | 0.5 |
|  |  |  |  |  |  |  |  |  |  |  |  |
| **δ** | **Deltaproteobacteria** |  |  |  |  |  |  |  |  |  |  |
| My | Myxococcales |  |  |  |  |  |  |  |  |  |  |
| Ana | Anaeromyxobacter | 0.05 | 0.15 | 0.15 | 0.3 | 0.2 | 0 | 0.4 | 0.2 | 0.2 | 0.2 |
| Bd | Bdellovibrio | 0.25 | 0.3 | 0.65 | 0.5 | 0.4 | 0.4 | 0.5 | 0.4 | 0.4 | 0.5 |
| Geob | Geobacter | 0 | 0 | 0.05 | 0.2 | 0.1 | 0 | 0.65 | 0.6 | 0.47 | 0.4 |
| GR | GR-WP33-30 unclas. | 0.75 | 1.05 | 1.35 | 1.3 | 0.4 | 0.1 | 0.05 | 0.1 | 0.03 | 0 |
| Hal | Haliangium | 0.75 | 0.65 | 1.0 | 0.97 | 0.9 | 0.1 | 0 | 0 | 0 | 0 |
| Hya | Hyalangium | 0.05 | 0.05 | 0.1 | 0.23 | 0.1 | 0.85 | 0.25 | 0.05 | 0.03 | 0.2 |
| My 0319 | Myxococcales 0319-6G20 unclas. | 0.55 | 0.45 | 0.5 | 0.33 | 0.25 | 0.15 | 0.05 | 0 | 0 | 0 |
| δ Prot und | Proteobacteri unclas. | 0.8 | 0.75 | 0.8 | 0.8 | 0.6 | 0.2 | 0.5 | 0.15 | 0.1 | 0.3 |
|  |  |  |  |  |  |  |  |  |  |  |  |
| **Acido** | **Acidobacteria (Phylum)** |  |  |  |  |  |  |  |  |  |  |
| Holo | Holophagaceae |  |  |  |  |  |  |  |  |  |  |
| Ac | Acidobacteriales |  |  |  |  |  |  |  |  |  |  |
| Acbia | Acidobacteriaceae | 0.85 | 1.35 | 0.85 | 0.86 | 0.7 | 0.05 | 0.1 | 0.1 | 0.03 | 0.1 |
| Aci und | Acidobacteria unclas. | 0.05 | 0.05 | 0 | 0 | 0.1 | 0 | 0 | 0 | 0 | 0 |
| Geot | Geothrix | 0 | 0 | 0.85 | 4.5 | 2 | 0 | 1.3 | 12.0 | 11.2 | 10 |
| SGs | Subgroups 2, 4, 5, 6, 11, 17, 25 | 20.2 | 20.9 | 16.5 | 15.2 | 2.6 | 1.45 | 1.55 | 1.1 | 0.3 | 0 |
| SG 7 | Subgroup 7 unclas. | 1.85 | 2.05 | 2.4 | 2.2 | 2 | 0.3 | 0.15 | 0.05 | 0.03 | 0 |
| SG 10 | Subgroup 10 unclas. | 0.05 | 0 | 0.05 | 0 | 0.5 | 0 | 0 | 0 | 0 | 0 |
| SG 22 | Subgroup 22 unclas. | 0.3 | 0.7 | 0.5 | 0.5 | 2 | 0 | 0 | 0 | 0 | 0 |
|  |  |  |  |  |  |  |  |  |  |  |  |
| **Actino** | **Actinobacteria (Phylum)** |  |  |  |  |  |  |  |  |  |  |
| Acidi | Acidimicrobidae |  |  |  |  |  |  |  |  |  |  |
| Act | Actinobacteridae |  |  |  |  |  |  |  |  |  |  |
| Cory | Corynebacteriales |  |  |  |  |  |  |  |  |  |  |
| Fra | Frankiales |  |  |  |  |  |  |  |  |  |  |
| Mi | Micrococcaceae |  |  |  |  |  |  |  |  |  |  |
| Prop | Propionibacteriales |  |  |  |  |  |  |  |  |  |  |
| Str | Streptomycetaceae |  |  |  |  |  |  |  |  |  |  |
| The | Thermoleophilales |  |  |  |  |  |  |  |  |  |  |
| Act und | Actinobacteria unclas. | 0.5 | 0.35 | 0.15 | 0.17 | 0.6 | 0.15 | 0.05 | 0 | 0 | 0 |
| Ales und | Acidimicrobiales unclas. | 0.67 | 0.4 | 0.5 | 0.5 | 0.5 | 0.1 | 0 | 0 | 0 | 0 |
| Arth | Arthobacter | 0.2 | 0.1 | 0.05 | 0.1 | 0.1 | 0.85 | 0.7 | 0.2 | 0.17 | 0.4 |
| Bla | Blastococcus | 0.15 | 0.05 | 0.1 | 0.03 | 0.1 | 0 | 0 | 0 | 0 | 0 |
| Cl 500 | Cl 500-29 | 0.85 | 1.05 | 0.65 | 0.43 | 0.6 | 0.1 | 0 | 0 | 0 | 0 |
| Gaie | Gaiella | 1.35 | 1.1 | 0.85 | 0.6 | 0.7 | 0.15 | 0.05 | 0.05 | 0.07 | 0 |
| Iam | Iamiaceae | 0.4 | 0.2 | 0.25 | 0.2 | 0.3 | 0.1 | 0 | 0 | 0 | 0 |
| Mar | Marmoricola | 0.3 | 0.05 | 0.1 | 0.18 | 0.2 | 0.1 | 0 | 0 | 0 | 0 |
| MB | MB-A2-108 | 0.4 | 0.3 | 0.15 | 0.2 | 0.3 | 0 | 0 | 0 | 0 | 0 |
| Myc | Mycobacterium | 0.3 | 0.2 | 0.15 | 0.2 | 0.2 | 0.1 | 0 | 0 | 0 | 0 |
| Nak | Nakamurella | 0.15 | 0.1 | 0.1 | 0.06 | 0.1 | 0.1 | 0.05 | 0 | 0 | 0 |
| Nor | Nocardioides | 0.2 | 0.1 | 0.1 | 0.1 | 0.1 | 0.15 | 0 | 0 | 0 | 0 |
| Ory | Oryzihumus | 0.2 | 0.25 | 0.1 | 0.2 | 0.2 | 0.1 | 0 | 0 | 0 | 0 |
| Stm | Streptomyces | 0.4 | 0.25 | 0.1 | 0.2 | 0.3 | 0.1 | 0 | 0 | 0 | 0 |
| Terr | Terrabacter | 0.15 | 0.15 | 0.05 | 0.06 | 0.1 | 0.25 | 0.2 | 0.1 | 0.1 | 0 |
|  |  |  |  |  |  |  |  |  |  |  |  |
| **Arma** | **Armatimonadetes (Phylum)** |  |  |  |  |  |  |  |  |  |  |
| Arma und | Armatimonadetes unclas. | 0.25 | 0.3 | 0.5 | 0.33 | 0.4 | 0.05 | 0 | 0 | 0 | 0 |
| Cht | Chthonomonas | 0.05 | 0.05 | 0.1 | 0.13 | 0.1 | 0 | 0 | 0 | 0 | 0 |
| Cht und | Chthonomonadaceae unclas. | 0.1 | 0.1 | 0.1 | 0.13 | 0.1 | 0 | 0 | 0 | 0 | 0 |
|  |  |  |  |  |  |  |  |  |  |  |  |
| **Can Div** | **Candidate Division (Phylum)** |  |  |  |  |  |  |  |  |  |  |
| OD1 | Candidate division OD1 unclas. | 0.1 | 0.3 | 0.65 | 0.23 | 0.4 | 0 | 0 | 0 | 0 | 0 |
| TM7 | Candidate division TM7 unclas. | 0.25 | 0.15 | 0.4 | 0.47 | 0.3 | 0.18 | 0.19 | 1.05 | 1.5 | 0 |
| WS3 | Candidate division WS3 unclas. | 1.05 | 1.5 | 1.4 | 0.8 | 1.0 | 0.05 | 0.05 | 0 | 0 | 1.5 |
|  |  |  |  |  |  |  |  |  |  |  |  |
| **Chloro** | **Chloroflexi (Phylum)** |  |  |  |  |  |  |  |  |  |  |
| Ana und | Anaerolineaceae |  |  |  |  | 0.1 |  |  |  |  | 0 |
| Chl und | Chloroflexales KD-96 | 2.25 | 1.85 | 0.95 | 1.1 | 0.1 | 0.2 | 0.05 | 0.1 | 0.06 | 0 |
| IG und | IG 30-FI-CM66 unclas. | 0.25 | 0.4 | 0.3 | 0.3 | 0.3 | 0.05 | 0 | 0 | 0 | 0 |
| P2 | P2-IIE | 0.05 | 0.1 | 0.15 | 0.13 | 0.2 | 0 | 0 | 0 | 0 | 0 |
| Ros | Roseiflexus | 0.6 | 0.25 | 0.2 | 0.3 | 0.3 | 0.1 | 0 | 0 | 0 | 0 |
| S085 | S085 unclas. | 0.2 | 0.15 | 0.1 | 0.18 | 0.15 | 0 | 0 | 0 | 0 | 0 |
| TK | TK-10 | 0.3 | 0.2 | 0.4 | 0.3 | 0.3 | 0 | 0 | 0 | 0 | 0 |
|  |  |  |  |  |  |  |  |  |  |  |  |
| **Bactes** | **Bacteriodetes (Phylum)** |  |  |  |  |  |  |  |  |  |  |
| Ch | Chitinophagaceae |  |  |  |  |  |  |  |  |  |  |
| Fv | Flavobacteria |  |  |  |  |  |  |  |  |  |  |
| Sph | Sphingobacteriia |  |  |  |  |  |  |  |  |  |  |
| Sles | Sphingobacteriales |  |  |  |  |  |  |  |  |  |  |
| Bac und | Bacteriodetes unclas. | 0.1 | 0.1 | 0.1 | 0.06 | 0.2 | 0.1 | 0.1 | 0.1 | 0.15 | 0 |
| Ch und | Chitinophagaceae unclas. | 3.55 | 2.25 | 2.35 | 1.2 | 1.5 | 0.8 | 0.8 | 0.2 | 0.1 | 0.2 |
| Chit | Chitinophaga | 0.15 | 0.1 | 0.1 | 0.13 | 0.1 | 0.25 | 0.2 | 0.05 | 0.07 | 0.1 |
| Env | OPS_17 unclas. | 0.4 | 0.55 | 1.15 | 0.45 | 0.5 | 0.05 | 0.05 | 0.05 | 0.07 | 0 |
| Ferr | Ferruginibacter | 0.45 | 0.2 | 0.3 | 0.17 | 0.3 | 0.25 | 0.15 | 0.05 | 0 | 0.05 |
| Fla | Flavobacterium | 0.35 | 0.35 | 0.75 | 0.77 | 0.6 | 31.5 | 9.95 | 3.2 | 2.4 | 10.7 |
| Fsol | Flavisolibacter | 0.15 | 0.1 | 0.15 | 0.2 | 0.1 | 0.8 | 1.95 | 0.45 | 0.47 | 0.6 |
| Pal | Paludibacter | 0 | 0 | 0.1 | 0.1 | 0.5 | 0 | 0 | 0.65 | 1.03 | 0.5 |
| Sap | Saprospiraceae unclas. | 0.55 | 0.4 | 0.3 | 0.1 | 0.2 | 0.05 | 0 | 0 | 0 | 0 |
| Sph und | Sphingobacteriia unclas. | 0.2 | 0.15 | 0.25 | 0.17 | 0.2 | 0 | 0 | 0 | 0 | 0 |
| Spo | Sporophytophaga | 0.05 | 0.15 | 0.15 | 0.15 | 0.2 | 0 | 0 | 0 | 0 | 0 |
| Terr | Terrimonas | 0.3 | 0.2 | 0.2 | 0.1 | 0.2 | 0.05 | 0.05 | 0 | 0 | 0 |
| WCH | B1-32 unclas. | 0 | 0 | 0.15 | 1.3 | 0.5 | 0 | 0.15 | 6.1 | 5.7 | 4 |
|  |  |  |  |  |  |  |  |  |  |  |  |
| **Fir** | **Firmicutes (Phylum)** |  |  |  |  |  |  |  |  |  |  |
| CB | Class Bacillus |  |  |  |  |  |  |  |  |  |  |
| CClo | Class Clostridium |  |  |  |  |  |  |  |  |  |  |
| An | Anaerosporobacter | 0 | 0 | 0.1 | 0.4 | 0.3 | 0.05 | 1.3 | 1.75 | 0.9 | 0.3 |
| Ba | Bacillus | 0.15 | 0.2 | 0.15 | 0.2 | 0.2 | 0.55 | 0.8 | 0.2 | 0.07 | 0.5 |
| Clo und | Clostridia unclas. | 0 | 0 | 0 | 0 | 0 | 0.1 | 0.1 | 0.1 | 0.1 | 0.3 |
| Al und | Unclassified order | 0.15 | 0.2 | 0.15 | 0.2 | 0 | 0.55 | 0.8 | 0.2 | 0.07 | 0.3 |
| Clo | Clostridium sensu stricto | 0 | 0 | 0 | 0 | 0 | 0.7 | 3.1 | 1.05 | 0.08 | 2 |
| Fon | Fonticella | 0 | 0 | 0.05 | 0.03 | 0.1 | 0.05 | 0.35 | 0.55 | 0.4 | 0.5 |
| Gr | Gracilibacteraceae | 0 | 0 | 0 | 0.23 | 0.1 | 0 | 0.1 | 0.4 | 0.37 | 0.5 |
| Na | Natranaerovirga | 0 | 0 | 0 | 0.1 | 0 | 0 | 0 | 0.05 | 0.43 | 0.3 |
| Ru | Ruminococcaceae | 0 | 0 | 0.05 | 0.13 | 0.1 | 0 | 0.1 | 0.75 | 0.73 | 0.7 |
| Ru und | Ruminococcaceae unclas. | 0 | 0 | 0.2 | 0.77 | 0.3 | 0 | 0.05 | 2.2 | 3.2 | 2.65 |
| Se | Sedimentibacter | 0 | 0 | 0.05 | 0.07 | 0 | 0 | 0 | 0.25 | 0.37 | 0.2 |
| Tr | Trichococcus | 0 | 0 | 0.05 | 0.23 | 0.1 | 0 | 0 | 0.1 | 0.73 | 0.5 |
|  |  |  |  |  |  |  |  |  |  |  |  |
| **Gem** | **Gemmatimonadetes (Phylum)** |  |  |  |  |  |  |  |  |  |  |
| Gas | Gemmatinomas | 0.8 | 0.8 | 0.7 | 0.53 | 0.7 | 0.1 | 0.1 | 0 | 0 | 0.03 |
| Gem und | Gemmatimonadaceae unclas. | 2.65 | 3.2 | 3.7 | 2.8 | 3.0 | 0.35 | 0.15 | 0.1 | 0.1 | 0.2 |
|  |  |  |  |  |  |  |  |  |  |  |  |
| **Nit** | **Nitrospirae (Phylum)** |  |  |  |  |  |  |  |  |  |  |
| Ni | Nitrospira | 0.65 | 0.8 | 0.95 | 0.8 | 0.8 | 0.05 | 0.05 | 0 | 0 | 0 |
| Ni und | 0319-6A21 | 1.7 | 2.4 | 2.2 | 2.0 | 2.0 | 0.15 | 0.1 | 0.1 | 0.03 | 0.1 |
|  |  |  |  |  |  |  |  |  |  |  |  |
| **Pla** | **Planctomycetes (Phylum)** |  |  |  |  |  |  |  |  |  |  |
| WD und | Panctomycetes WD2101 unclas. | 0.65 | 0.85 | 0.95 | 0.67 | 0.7 | 0.05 | 0.1 | 0.05 | 0.06 | 0.1 |
|  |  |  |  |  |  |  |  |  |  |  |  |
| **Ver** | **Verrucomicrobia (Phylum)** |  |  |  |  |  |  |  |  |  |  |
| DA | DA 101 soil group unclas. | 0.8 | 0.85 | 0.95 | 0.87 | 0.7 | 0.05 | 0 | 0.05 | 0.06 | 0 |
| OPB | OPB 35 soil group unclas. | 0.5 | 0.65 | 0.75 | 0.57 | 0.5 | 0.05 | 0 | 0 | 0.03 | 0 |
|  |  |  |  |  |  |  |  |  |  |  |  |
| **Arch** | **Archaea (Domain)** |  |  |  |  |  |  |  |  |  |  |
| **Tha** | **Thaumarchaeota (Phylum)** |  |  |  |  |  |  |  |  |  |  |
| SCG | Soil Crenarchaeotic Group | 3.95 | 4.7 | 3.95 | 1.47 | 3.3 | 0.1 | 0.1 | 0.1 | 0.1 | 0.1 |

und, unclas., unclassified
