# Supplementary material for: Disruption of microbial community composition and identification of plant growth promoting microorganisms after exposure of soil to rapeseed-derived glucosinolates
Source: PLoS One. 2018 Jul 3;13(7):e0200160. doi: 10.1371/journal.pone.0200160 (PMC6029813; doi:10.1371/journal.pone.0200160)
Supplement: S4 Table — (DOCX) [file pone.0200160.s014.docx]

| **S4 Table. Fungal taxonomical groups detected in soil samples.** | | | | | | | | | |
| --- | --- | --- | --- | --- | --- | --- | --- | --- | --- |
| *Abbre-viation* | *Taxonomic Group* | *Abundance*  *Control (%)* | | | | *Abundance*  *RS-EX (%)* | | | |
|  |  | 7 d | 14 d | 21 d | 28 d | 7 d | 14 d | 21 d | 28 d |
| **Asco** | **Ascomycota (Phylum)** |  |  |  |  |  |  |  |  |
| A und | Ascomycota family Incertae sedis | 0.2 | 0.2 | 0 | 0 | 0 | 0 | 0 | 0 |
| Acre | Acremonium | 0.2 | 0.2 | 0.2 | 0 | 0 | 0 | 0 | 0 |
| Ap | Apodus | 0.2 | 0.15 | 0 | 0.1 | 0 | 0 | 0 | 0 |
| Asco und | Ascomycota unclas. | 3.1 | 2.7 | 0.75 | 0.6 | 0.1 | 0 | 0 | 0 |
| Bio | Bionectriaceae unclas. | 0.75 | 0.4 | 0.2 | 0.2 | 0 | 0 | 0 | 0 |
| Chaem | Chaetomiaceae | 0.6 | 0.15 | 0.1 | 0 | 0 | 0 | 0 | 0 |
| Chae und | Chaetosphaeriaceae unclas. | 2.45 | 2.3 | 1.25 | 0.1 | 0.1 | 0.1 | 0.1 | 0.1 |
| Conio | Coniochaetaceae unclas. | 0.35 | 0.4 | 0.1 | 0.1 | 0 | 0 | 0 | 0 |
| Davi und | Davidiellaceae unclas. | 1.4 | 33.6 | 70.5 | 61 | 0 | 0.3 | 0.7 | 1 |
| Dev | Devriesia | 0.15 | 2.5 | 0.05 | 0.05 | 0 | 0 | 0 | 0 |
| Dot | Dothideomycetes unclas. | 0.15 | 0.35 | 0 | 0 | 0 | 0 | 0 | 0 |
| Ex | Exophiala | 1.35 | 0.9 | 0.2 | 0.15 | 0.05 | 0 | 0.1 | 0 |
| Fus | Fusarium | 4 | 4 | 1.3 | 2.2 | 5 | 4.75 | 1.3 | 0.8 |
| Ha | Haematonectria | 1.55 | 1.5 | 0.7 | 0.4 | 11.8 | 13 | 3 | 0.6 |
| Hel | Helotiales Incertae sedis | 0.9 | 0.25 | 0.55 | 0.01 | 0 | 0 | 0 | 0 |
| Hyp | Hypocreaceae unclas. | 0.35 | 0.35 | 0.25 | 0.15 | 0 | 0 | 0 | 0 |
| Ily | Ilyonectria | 1.5 | 1.8 | 0.1 | 0.1 | 0 | 0 | 0 | 0 |
| Las | Lasiosphaeriaceae | 0.35 | 1.2 | 0.1 | 0.1 | 0.2 | 0.15 | 0.05 | 0 |
| Leo und | Leotiomycetes unclas. | 0.35 | 0.15 | 0 | 0.15 | 0 | 0 | 0 | 0 |
| Mi | Microascaceae | 0.3 | 0.15 | 0.2 | 0.1 | 0 | 0 | 0 | 0 |
| Micr | Microdochium | 0.3 | 0.1 | 0 | 0.1 | 0 | 0 | 0 | 0 |
| Nec und | Nectriaceae unclas. | 0.6 | 0.9 | 0.4 | 0.3 | 0 | 0 | 0 | 0 |
| Neo | Neonectria | 0.3 | 0.2 | 0 | 0.1 | 0 | 0 | 0 | 0 |
| Pae | Paecilomyces | 0.2 | 0.15 | 0.15 | 0.1 | 0 | 0 | 0 | 0 |
| Ph | Phialocephala | 0.55 | 0.5 | 0.4 | 0 | 0 | 0 | 0 | 0 |
| Phi | Phialophora | 1.7 | 0.15 | 0 | 0 | 0 | 0 | 0 | 0 |
| Pl und | Pleosporales family Incertae sedis | 1.15 | 0.8 | 0.25 | 0.2 | 0 | 0 | 0 | 0 |
| Ple | Pleurophragmium | 0.35 | 0.2 | 0.15 | 0 | 0 | 0 | 0 | 0 |
| Plec und | Plectosphaerellaceae unclas. | 2.2 | 1.4 | 0.5 | 0.15 | 0.15 | 0 | 0 | 0 |
| Pleo und | Pleosporales unclas. | 0.25 | 0.35 | 0 | 0 | 0.1 | 0 | 0 | 0 |
| Pleo | Pleosporales | 0.15 | 0.15 | 0 | 0 | 0 | 0 | 0 | 0 |
| Pseu | Pseudeurotium | 2 | 1.4 | 0.55 | 0.8 | 0.05 | 0.1 | 0.15 | 0.2 |
| Pseuda | Pseudaleuria | 2.35 | 3.05 | 0.05 | 0.65 | 45.5 | 5.9 | 4.85 | 25 |
| Pyre | Pyrenochaetopsis | 0.8 | 0.55 | 0.25 | 0 | 0 | 0 | 0 | 0 |
| Pyro und | Pyronemataceae unclas. | 0.1 | 0.45 | 0.5 | 0 | 0.8 | 2 | 3.7 | 0 |
| Sor | Sordariomycetes |  |  |  |  |  |  |  |  |
| Sor und | Sordariomycetes unclas. | 0.7 | 0.35 | 0.45 | 0.3 | 0.1 | 0 | 0 | 0 |
| Sta | Staphylotrichum | 0.65 | 0.7 | 0.45 | 0.35 | 7.3 | 4.5 | 1.3 | 0.4 |
| Te | Tetracladium | 2.5 | 1.6 | 0.55 | 0.05 | 0 | 0.05 | 0 | 0 |
| Trichod | Trichoderma | 1 | 0.8 | 0.3 | 0.15 | 0.2 | 0.1 | 0.3 | 0.1 |
|  |  |  |  |  |  |  |  |  |  |
| **Basi** | **Basidiomycota (Phylum)** |  |  |  |  |  |  |  |  |
| Cry | Cryptococcus | 3.1 | 1.25 | 1.2 | 0.65 | 0 | 0 | 0 | 0 |
| Min | Minimedusa | 0.35 | 0.05 | 0 | 0 | 0 | 0 | 0 | 0 |
| Rho | Rhodotorula | 0.1 | 0.05 | 0.05 | 0.05 | 0 | 0 | 0 | 0 |
| Spo | Sporidiobolales unclas. | 0.2 | 0.05 | 0.1 | 0 | 0 | 0 | 0 | 0 |
| Tre | Tremellomycetes unclas. | 1.4 | 0.7 | 0.8 | 0.55 | 0 | 0 | 0 | 0 |
| Trich | Trichosporon | 0.6 | 1.45 | 4.6 | 21 | 0.65 | 8.3 | 85 | 95 |
|  |  |  |  |  |  |  |  |  |  |
| **Zyg** | **Zygomycota (Phylum)** |  |  |  |  |  |  |  |  |
| Mor | Mortierella | 47.1 | 21 | 9 | 6.25 | 29.5 | 7.9 | 1.65 | 0.6 |

und, unclass., unclassified
