# Supplementary material for: Disruption of microbial community composition and identification of plant growth promoting microorganisms after exposure of soil to rapeseed-derived glucosinolates
Source: PLoS One. 2018 Jul 3;13(7):e0200160. doi: 10.1371/journal.pone.0200160 (PMC6029813; doi:10.1371/journal.pone.0200160)
Supplement: S5 Table — (DOCX) [file pone.0200160.s015.docx]

**S5 Table. Primers used for taxonomic classification of cultivable microorganisms.**

|  | | | |
| --- | --- | --- | --- |
| Primer | Name | Sequence (5'-3') |  |
| bn2635 | 1492R | CGGTTACCTTGTTACGACTT | 16S-forward |
| bn2636 | 27f | AGAGTTTGATCMTGGCTCAG | 16S-reverse |
| bn2429 | ITS5 ITS | GGAAGTAAAAGTCGTAACAAGG | ITS-forward |
| bn2430 | LR6 ITS | CGCCAGTTCTGCTTACC | ITS-reverse |
| bn2431 | LR12R IGS1 | CTGAACGCCTCTAAGTCAGAA | IGS1-forward |
| bn2432 | 5SR2 IGS1 | CTCCBTGGTACTAACYGAGCG | IGS1-reverse |
