# Supplementary material for: Disruption of microbial community composition and identification of plant growth promoting microorganisms after exposure of soil to rapeseed-derived glucosinolates
Source: PLoS One. 2018 Jul 3;13(7):e0200160. doi: 10.1371/journal.pone.0200160 (PMC6029813; doi:10.1371/journal.pone.0200160)
Supplement: S6 Table — (DOCX) [file pone.0200160.s016.docx]

**S6 Table. Sequences of 16S RNA and ITS amplicons from isolated bacteria and fungi, respectively.**

| **Bacteria** |
| --- |
| *Acinetobacter kooki*  GtAGCGTCCTCCTTGCGGTTAGACTACCTACTTCTGGTGCAACAAACTCCCATGGTGTGACGGGCGGTGTGTACAAGGCCCGGGAACGTATTCACCGCGGCATTCTGATCCGCGATTACTAGCGATTCCGACTTCATGGAGTCGAGTTGCAGACTCCAATCCGGACTACGATCGGCTTTTTGAGATTAGCATCCTATCGCTAGGTAGCAACCCTTTGTACCGACCATTGTAGCACGTGTGTAGCCCTGGTCGTAAGGGCCATGATGACTTGACGTCGTCCCCGCCTTCCTCCAGTTTGTCACTGGCAGTATCCTTAAAGTTCCCGGCATGACCCGATGGCAAGTAAGGAAAAGGGTTGCGCTCGTTGCGGGACTTAACCCAACATCTCACGACACGAGCTGACGACAGCCATGCAGCACCTGTATCAGAGTTCCCGAAGGCACCAATCCATCTCTGGAAAGTTCTCTGTATGTCAAGACCAGGTAAGGTTCTTCGCGTTGCATCGAATTAAACCACATGCTCCACCGCTTGTGCGGGCCCCCGTCAATTCATTTGAGTTTTAGTCTTGCGACCGTACTCCCCAGGCGGTCTACTTATCGCGTTAGCTGCGCCACTAAAGCCTCAAAGGCCCCAACGGCTAGTAGACATCGTTTACGGCATGGACTACCAGGGTATCTAATCCTGTTTGCTCCCCATGCTTTCGTACCTCAGCGTCAGTATTAGGCcAgAtGGCTGCCTTCgCCATCGGTATTCCTCCAGATCTCTACGCATTTCACCGCTACACCtggAATTCTAcCATCCTCTCCCATACTCtAgCCTCCCAGTATCgAAtGcAATTCCtnAgTTAAgCTCagGgnnTTCaCATCCgACTTaAAagcCGCCtACGCaCGCTtnncgCCcaGTAAATcCnAttaacGCTCGCACCCtctGTAttanCG |
| *Aminobacter aminovorans*  gGtnncCTGCCTCCTTGCGGTTAGCaCAGTGCCTTCGGGTAAAACCAACTCCCATGGTGTGACGGGCGGTGTGTACAAGGCCCGGGAACGTATTCACCGCAGCATGCTGATCTGCGATTACTAGCGATTCCAACTTCATGCACTCGAGTTGCAGAGTGCAATCCGAACTGAGATGGCTTTTGGAGATTAGCTCGACCTCGCGGTCTCGCTGCCCACTGTCACCACCATTGTAGCACGTGTGTAGCCCAGCCCGTAAGGGCCATGAGGACTTGACGTCATCTTCACCTTCCTCGCGGCTTATCACCGGCAGTCCCCTTAGAGTGCCCAACTTAATGATGGCAACTAAGGGCGAAGGTTGCGCTCGTTGCGGGACTTAACCCAACATCTCACGACACGAGCTGACGACAGCCATGCAGCACCTGTCACCGGTCCAGCCGAACTGAAGGGATCCATCTCTGGAAACCGCGACCGGGATGTCAAGGGCTGGTAAGGTTCTGCGCGTTGCTTCgAATTAAACCACATGCTCCACCGCTTGTGCGGGCCCCCGTCAATTCCTTTGAGTTTTAATCTTGCGACCGTACTCCCCAGGCGGAGAGCTTAATGCGTTAGCTGCGCCACCGACAAGTAAACTTGCCGACGGCTAGCTCTCATAGTTtACGGCGTGGACTACCagGGTATCTAATCcTGTTTGCTCCCCACGCTTTCGCACCTCAncgncaGTACCGAgCCAGTGAgCCgCCTTCGCCACTGGtGTTCCTCCgAATAtCTACnAatTtCAcCTCtACACTCggAATTCCAcTCacCtcTCTcGgAntCgAgAtngCcantATnaaagGcAGTtCcngGGtTGAnCCC |
| *Bacillus aryabhattai*  AnTCcaCCGACTTCGGGTGTtacAAACTCTCGTGGTGTGACGGGCGGTGTGTACAAGGCCCGGGAACGTATTCACCGCGGCATGCTGATCCGCGATTACTAGCGATTCCAGCTTCATGTAGGCGAGTTGCAGCCTACAATCCGAACTGAGAATGGTTTTATGGGATTGGCTTGACCTCGCGGTCTTGCAGCCCTTTGTACCATCCATTGTAGCACGTGTGTAGCCCAGGTCATAAGGGGCATGATGATTTGACGTCATCCCCACCTTCCTCCGGTTTGTCACCGGCAGTCACCTTAGAGTGCCCAACtAaaTGCTGGCAACTAAGATCAAGGGTTGCGCTCGTTGCGGGACTTAACCCAACATCTCACGACACGAGCTGACGACAACCATGCACCACCTGTCACTCTGTCCCCCGAAGGGGAACGCTCTATCTCTAGAGTTGTCAGAGGATGTCAAGACCTGGTAAGGTTCTTCGCGTTGCTTCGAATTAAACCACATGCTCCACCGCTTGTGCGGGCCCCCGTCAATTCCTTTGAGTTTCAGTCTTGCGACCGTACTCCCCAGGCGGAGTGCTTAATGCGTTAGCTGCAGCACTAAAGGGCGGAAACCCTCTAACACTTAGCACTCATCGTTTACGGCGTGGACTACCAGGGTATCTAATCCTGTTTGCTCCCCACGCTTTCGCGCCTCAGCGTCAGTTACAGACCAAAAAGCCGCCTTCGCCACTGGTGTTCCTCCACATCTCTACGCATTTCACCGCTACACGTGGAATTCCGCTTTTCTCTTCTGCACTCAAGTTCCCCAGTTTCCAATGACCCTCCACGGTTGAGCCGtgggCTTTCACATCAGACTTAAGAAaCCGCCTGCGCGCGCTTTACGCCCAATAATTCCGGATAACGCTTGCCACCTACGTATTACcGcggCTGCTGGCAcntaTnnAGccntGGCTTTCTGGTTaaGTACCGncaaGgTAcgaGcnnTtACtctcgtACttgTTCTtCCcTAAcaacaaa |
| *Bacillus cereus*  anGcGGcTGGCTCcnTAAAGGTTACCCCACCGACTTCGGGTGTTACAAACTCTCGTGGTGTGACGGGCGGTGTGTACAAGGCCCGGGAACGTATTCACCGCGGCATGCTGATCCGCGATTACTAGCGATTCCAGCTTCATGTAGGCGAGTTGCAGCCTACAATCCGAACTGAGAACGGTTTTATGAGATTAGCTCCACCTCGCGGTCTTGCAGCTCTTTGTACCGTCCATTGTAGCACGTGTGTAGCCCAGGTCATAAGGGGCATGATGATTTGACGTCATCCCCACCTTCCTCCGGTTTGTCACCGGCAGTCACCTTAGAGTGCCCAACTTAATGATGGCAACTAAGATCAAGGGTTGCGCTCGTTGCGGGACTTAACCCAACATCTCACGACACGAGCTGACGACAACCATGCACCACCTGTCACTCTGCTCCCGAAGGAGAAGCCCTATCTCTAGGGTTTTCAGAGGATGTCAAGACCTGGTAAGGTTCTTCGCGTTGCTTCGAATTAAACCACATGCTCCACCGCTTGTGCGGGCCCCCGTCAATTCCTTTGAGTTTCAGCCTTGCGGCCGTACTCCCCAGGCGGAGTGCTTAAtGCGTTAACTTCAGCACTAAAGGGCGGAAACCCTCTAACACTTAGCACTCATCGTTTACGGCGTGgACTACCAgGGTATCTAATCCTGTTTGCTCCCCACGCTTTCnCGCCTCAgtGTCAGtTACAGACCAnAAAGTCnCCTTCgCCACTGGnGTTCCtCcnTATCTCTACgCATTTcACCGcTACaCATGgAatTCcACtTTTCCTCtTCTGCACTCAAGtCTCCCAgtTTTCCnAtGAcccTCCAcnG |
| *Bacillus megaterium*  caCCGACTTCGGGTGTTACAAACTCTCGTGGTGTGACGGGCGGTGTGTACAAGGCCCGGGAACGTATTCACCGCGGCATGCTGATCCGCGATTACTAGCGATTCCAGCTTCATGTAGGCGAGTTGCAGCCTACAATCCGAACTGAGAATGGTTTTATGGGATTGGCTTGACCTCGCGGTCTTGCAGCCCTTTGTACCATCCATTGTAGCACGTGTGTAGCCCAGGTCATAAGGGGCATGATGATTTGACGTCATCCCCACCTTCCTCCGGTTTGTCACCGGCAGTCACCTTAGAGTGCCCAACtaaATGCTGGCAACTAAGATCAAGGGTTGCGCTCGTTGCGGGACTTAACCCAACATCTCACGACACGAGCTGACGACAACCATGCACCACCTGTCACTCTGTCCCCCGAAGGGGAACGCTCTATCTCTAGAGTTGTCAGAGGATGTCAAGACCTGGTAAGGTTCTTCGCGTTGCttnnnatTAAACCACATGCTCCACCGCTTGTGCGGGCCCCCGTCAATTCCTTtgnnnttcagtctTGcGACCGTACTCCcc |
| *Bacillus amylolique-faciens*  TCnGcgGCtGGCTCcnTAAAGGTTACCTCACCGACTTCGGGTGTTACAAACTCTCGTGGTGTGACGGGCGGTGTGTACAAGGCCCGGGAACGTATTCACCGCGGCATGCTGATCCGCGATTACTAgCGATTCCAGCTTCACGCAGTCgAgTTGCAGACTGCGAtCCgAACTGAGAACAGATTTGTGGGATTGGCTTAACCTCGCGGTTTCGCTGCCCTTTGTTCTGTCCATTGTAGCACGTGTGTAGCCCAGGTCATAAGGGGCATGATGATTTGACGTCATCCCCACCTTCCTCCGGTTTGTCACCGGCAGTCACCTTAGAGTGCCCAACTGAATGCTGGCAACTAAGATCAAGGGTTGCGCTCGTTGCGGGACTTAACCCAACATCTCACGACACGAGCTGACgACAACCATGCACCACCTGTCACTCTGCCCCCGAAGGGGACGTCCTATCtcnnngATTGTCAgAgGATGtcaagACcnggta |
| *Bacillus mycoides*  CnAAAnGGgttaCcccaCCGACTTCGGGTGTtacAAACTCTCGTGGTGTGACGGGCGGTGTGTACAAGGCCCGGGAACGTATTCACCGCGGCATGCTGATCCGCGATTACTAGCGATTCCAGCTTCATGTAGGCGAGTTGCAGCCTACAATCCGAACTGAGAACGGTTTTATGAGATTAGCtCcACCTCGCGGTCTTGCAGCTCTTtGTACCGTCCATtgnaGcncGTGTGTAGCCCAGGTCATAAGGGGCATgatGATTTGACGTCATCCCCACCTTCCTCCGGTTTGTCACCGGCAGTCaCcTTAGAGTGCCCAACTTAATGATGGCAACTAAGATCAAGGGTTGCGCTCGTTGCGGGACTTAACCCAACATCTCACGACACGAGCTGACGACAACCATgcnnACCTGTCaCTCTGCCCCCGAAGGGGAAGCCCTATCtcaGGGTTGtcgaGGATgTCAAGACCTggtAAGGTTcttCGCGTtgcTT |
| *Chitinophaga niastensis*  gGcGGTTcCTTGCGGTTGCCGACTTCaGGTCCCCCCGGCTTTCaTGGCTTGACGGGCGGTGTGTACAAGGTCCGGGAACGTATTCACCGTATCATTGCTGATATACGATTACTAGCGATTCCAGCTTCATGAGGTCGAGTTGCAGACCTCAATCCGAACTGAGATGGGATTTTTGAGATTAGCAGCCTGTTACCAGGTAGCAGCCCTTTGTTCCCACCATTGTAGCACGTGTGTAGCCCTGGGCATAAAGGCCATGATGACTTGACATCATCCCCTCCTTCCTCGCGTCTTACGACGGCAGTTTCACTAGAGTTCCCACCATTACGTGCTGGCAACTAGTGATAGGGGTTGCGCTCGTTGCGGGACTTAACCCAACACCTCACGGCACGAGCTGACGACAGCCATGCAGCACCTTACAAGAAGTGTATTGCTACAAAGACAGCTTTCACCATCGGTCTTCCTGCATTCTAGCCCAGGTAAGGTTCCTCGCGTATCATCgAATTAAACCACATGCTCCACCGCTTGTGCGGACCCCCGTCAATTCCTTTGAntTTCAACCTTGCGGTCGTACTTCCCAGGTGGATTACTTAATGCTTTCgCTCAnACACTTGCTGTGTATCGCAAATGTCgAGTAATCnTCGTTTAgGGCgTGgACTACCAgGGTATCtaantccTGtTTGATCCCCaCgctTtCGTGccncanc |
| *Enterobacter sp.*  tcnnaAgGGcCatGAtgACTTGAcgtCaTCCCCacCtTCCtCCagtTTatcaCTGGcantCTCCTTTgAntTccCgnncggACcgntggcaAcAaanganAAGGGTTGCgCTCGTTGCgggACTtAACCCAACATTTCAcaACaCAAgcTgAnnACAgcca |
| *Lysinibacillus fusiformis*  tcnGcGGCTGGCTCcnTAAAGGTTACCCCACCGACTTCGGGTGTTACAAACTCTCGTGGTGTGACGGGCGGTGTGTACAAGGCCCGGGAACGTATTCACCGCGGCATGCTGATCCGCGATTACTAGCGATTCCGGCTTCATGTAGGCGAGTTGCAGCCTACAATCCGAACTGAGAACGACTTTATCGGATTAGCTCCCTCTCGCGAGTTGGCAACCGTTTGTATCGTCCATTGTAGCACGTGTGTAGCCCAGGTCATAAGGGGCATGATGATTTGACGTCATCCCCACCTTCCTCCGGTTTGTCACCGGCAGTCACCTTAGAGTGCCCAACTAAATGATGGcaACTAAGATCAAGGGTTGCGCTCGTTGCGGGACTTAACCCAACATCTCACGACACGAGCTGACGACAACCATGCACCACCTGTCACCGTTGTCCCCGAAGGGAAAACCATATCTCTACAGTGGTCAACGGGATGTCAAGACCTGGTAAGGTTCTTCGCGTTGCTTCGAATTAAACCACATGCTCCACCGCTTGTGCGGGCCCCCGTCAATTCCTTTGAGTTTCAGTCTTGCGACCGTACTCCCCAGGCGGAGTGCTTAATGCgttnncTGCAGCACTAAgGGGCGgAAACCCCCTAncACTTAnCACTCaTCGtttACGGCgTgnAcTACCanGgtATCTaAaTCCtGTTTGcTCnccaCGCTTTCgCGCcTcnncgtC |
| *Lysinibacillus xylanilyticus*  AannGgttaCcccaCCGACTTCGGGTGTtacAAaCTCTCGTGGTGTGACGGGCGGTGTgtacAAGGCCCGGGAaCGTATTCACCGCGGCATGCTGATCCGCGATTACTAGCGATTCCGGCTTCATGTAGGCGAGTTGCAGCCTACAATCCGAActGAGAACGACTTTATCGGATTAGCTCCCTctCGCGAGTTGGCAACCgTttGTATCGTCCATTgtaGcncGTGTGTAGCCCAGGTCATAAGGGGCATGATGATTTGACGTCATCCCCACCTTCCTCCGGTTTGTCACCGGCAGTCACCTTAGAGTGCCCAACTAAATGATGGCAACTAAGATCAAGGGTTGCGCTCGTTGCGGGACTTAACCCAACATCtCACgACACGAGCTGACGACAACCATGCACCACCTGTCACCGTTGTCCCCGAAGGGAAaaCcATATCTCTACAGTGGTcnaCGGGATGTcangACCTGGTAaGgttCTTcnnGt |
| *Mycobacterium fortuitum*  tAntCgCagGTGTAgCGCGggTagATGTCCgCGGtGgcCcaCagcccgtTGTCATGCATGaCACTGCCCAGGGcGTCnAGCACCGACGTGCGGTAgTTCGGGAACAgCGCGTTCAACGCCGGCaCCCGcAGGTACAGCAATTGCgAAGTGCTGGcgAGGCTTCCGAGCAGCTGCACCATGGTGTCGGAGTTGTCgGCgAACAGGTTGTCGACGGAGTTCAACGCTCCCGGGGTCTGGTTCGTGAGGCGACGGAAGCCCTCGCGCATCTTGTTGATCCCGTCgAACGTCGAACTCAAATTGTCCGACGCCACCGCGACGCCGGCGTTCTTCTCCGACATCagGTTGAAgACCACGCGGCTGTTGCGCAgCACGCTCACCGTCTCGGGCAGCACCGAATCCagCGTCnACAGGAGGAACGTGCCgCCGTCgATGACgTCgGCCAACTTGcGGGGACCGGCTTGcgAcaTACTCAGTTcccGGCGGATCActtnnaGCTTGCCGACaTCnaCCTGGGACAgCgCGCCGTCGgcatCggngAGCAACTGgGccanGcTGACCGGCaccGnGGCCTTGCCcagGncgAtcacgCTGc |
| *Paenibacillus polymyxa*  tggctCcTtgcGgTTTtACCTCACCGACTTCGGGTGTTGTAAACTCTCGTGGTGTGACGGGCGGtgtgtACAAGACCCGGGAACGTATTCACCGCGGCATGCTGATCCGCGATTACTAGCAATTCCGACTTCATGTAGGCGAGTTGCAGCCTACAATCCGAACTGAGACCGGCTTTTCTAGGATTGGCTCCagaTCGctccTTCGCTTCCCGTTGTACCGGCCATTGTAGTACGTGTGTAGCCCAGGTCATAAGGGGCATGATGATTTGACGTCATCCCCACCTTCCTCCGGTTTGTCACCGGCAGTCTGCTTAGAGTGCCCAGCttgACCTGCTGGCAACTAAGCATAAGGGTTGCGCTCGTTGCGGGACTTAACCCAACATCTCACGACACGAGCTGACGACAACCATGCACCACCTGTCTCCTCTGTCCCGAAGGAAAGGTCTATCTCTAGACCGGTCagaGGGATGTCAAGACCTGGTAAGGTTCTTCGCGTTGCTTCGAATTAAACCACATACTCCACTGCTTGTGCGGGTCCCCGTCAATTCCTTTGAGTTTCAGTCTTGCGACCGTACTCCCCAGGCGGAATGCTTAATGTGTTAACTTCGGCACCAAGGGTATCGAAACCCCTAACACCTAGCATTCATCGTTTACGGCGTGGACTACCAGGGTATCTAATCCTGTTTGCTCCCCACGCTTTCGCGCCTCAGCGTCAGTTACAGCCCAGAGAGTCGCCTTCGCCACTGGTGTTCCTCCACATCTCTACGCATTTCACCGCTACACGTGGAATTCCACTCTCCTCTTCTGCACTCAAGCTCCCCAGTTTCCAGTGCGACCCGAAGTTGAGCCTCGGGATTAAACACCAGACTTAAAGAGCCGCCTGCGCGCGCTTTACGCCCAATAATTCCGGACAACGCTTGCCCCCTACGTATTACCGCGGCTGCTGGCACGTAGTTAGCCGGGGCTTTCTTCTCaGgTACCGTCACTCttnnaGCAGTTACTCTAcAanaCGTTCTTCCCTGGCaacaGAgcTTtaCGATCCGAAAaCCTTCATCACTCagncGGCGTTGCTccGTCAGGCTTtcgCCcATTGCGgnanaaTTCCCTActGCTGcctccnnt |
| *Pseudomonas frederikbergensis*  tcgcagggcgAGCTACAATGCAGTCGAGCGGCAGCACGGGTACTTGTACCTGGTGGCGAGCGGCGGACGGGTGAGTAATGCCTAGGAATCTGCACTGGTAGTGGGGGATAACGTTCGGAAACGAACGCTAATACCGCATACGTCCTACGGGAGAAAGCAGGGGACCTTCGGGCCTTGCGCTATCAGATGAGCCTAGGTCGGATTAGCTAGTTGGTGAGGTAATGGCTCACCAAGGCGACGATCCGTAACTGGTCTGAGAGGATGATCAGTCACACTGGAACTGAGACACGGTCCAGACTCCTACGGGAGGCAGCAGTGGGGAATATTGGACAATGGGCGAAAGCCTGATCCAGCCATGCCGCGTGTGTGAAGAAGGTCTTCGGATTGTAAAGCACTTTAAGTTGGGAGGAAGGGCAGTTACCTAATACGTAATTGTTTTGACTTACCGACAGAATAAGCACCGGCTAACTCTGTGCCAGCAGCCGCGGTAATACAGAGGGTGCAAGCGTTAATCGGAATTACTGGGCGTAAAGCGCGCGTAGGTGGTTCGTTAAGTTGGATGTGAAATCCCCGGGCTCAACCTGGGAACTGCATTCAAAACTGTCGAGCTAGAGTATGGTAGAGGGTGGTGGAATTTCCTGTGTAGCGGTGAAATGCGTAGATATAGGAAGGAACACCAGTGGCGAAGGCGACCACCTGGACTGATACTGACACTGAGGTGCGAAAGCGTGGGGAGCAAACAGGATTAGATACCCTGGTAGTCCACGCCGTAAACGATGTCAACTAGCCGTTGGGAGCCTTGAGCTCTTAGTGGCGCAGCTAACGCATTAAGTTGACCGCCTGGGGAGTACGGCCGCAAGGTTAAAACTCAAATGAATTTGACGGGGGCCCGCACAAGCGGTGGAGCATGTGGTTTAATTCGAAGCAACGCGAG |
| *Pseudomonas syringae*  gcgacaccgtggtaccgtCCCCCGAAGGTTAACTAGCTACTTTGGTGCAACCCACTCCCATGGTGTGACGGGCGGTGTGTACAAGGCCCGGGAACGTATTCACCGCGACATTCTGATTCGCGATTACTAGCGATTCCGACTTCACGCAGTCGAGTTGCAGACTGCGATCCGGACTACGATCGGTTTTATGGGATTAGCTCCACCTCGCGGCTTGGCAACCCTCTGTACCGACCATTGTAGCACGTGTGTAGCCCAGGCCGTAAGGGCCATGATGACTTGACGTCATCCCCACCTTCCTCCGGTTTGTCACCGGCAGTCTCCTTAGAGTGCCCACCATTACGTGCTGGTAACTAAGGACAAGGGTTGCGCTCGTTACGGGACTTAACCCAACATCTCACGACACGAGCTGACGACAGCCATGCAGCACCTGTCTCAATGTTCCCGAAGGCACCAATCCATCTCTGGAAAGTTCATTGGATGTCAAGGCCTGGTAAGGTTCTTCGCGTTGCTTCGAATTAAACCACATGCTCCACCGCTTGTGCGGGCCCCCGTCAATTCATTTGAGTTTTAACCTTGCGGCCGTACTCCCCAGGCGGTCAACTTAATGCGTTAGCTGCGCCACTAAGAGCTCAAGGCTCCCAACGGCTAGTTGACATCGTTTACGGCGTGGACTACCAGGGTATCTAATCCTGTTTGCTCCCCACGCTTTCGCACCTCAGTGTCAGTATCAGTCCAGGTGGTCGCCTTCGCCACTGGTGTTCCTTCCTATATCTACGCATTTCACCGCTACACAGGAAATTCCACCACCCTCTACCATACTCTAGCTCGACAGTTTTGAATGCAGTTCCC  AGGTTGAGCCCGGGGATTTCACATCCAACTTAACGAACCACCTACGCGCG  CTTTACGCCCAGTAATTCCGATTAACGCTTGCACcctctgtattaccgcg |
| *Shigella flexneri*  ccTCcCGaagGtTAAGCTAcCtACtTCTTTtgcaACCCAcTCCCATGGtGtGaCgGGCGGtGtGtaCAAGGCCCGGGAACGTATTCACCGTGGCATTCTGATCCACGATTACTAgCGATTCCGACTTCATGGAGTCgAgTTGCaGACTCCAATCCGGACTACGACGCACTTTATGAGGTCCGCTTGCTCTCGCGAGGTCGCTTCTCTTTGTATGCGCCATTGtAnCACGTGTGTAGCCCTGGTCGTAAGGGCCATGAtGACTTGACGTCATCCCCACCTTCCTCCAGTTTATCACTGGCAGTCTCCTTTGAgTTCCCGGCCGGACCGCTggcaacAAAgGATAAGGGTTGCGCTCgtTGCGGgACTTannccaacatttcacaaCacgAgCTGACGACAgCCATGcancAcctgtct |
| *Solibacillus silvestris*  gCAGTCGAGCGgnnttTTATTGGTGCTTGCACCTTtnnnttTTAGCGGCGGACGGGTGAGTAACACGTGGGTAACCTACCTTATAGATTGGGATAACTCCGGGAAACCGGGGCTAATACCGAATAATACTTTTTAACACATGTTTGAAAGTTGAAAGACGGTTTCGGCTGTCACTATAagaTGGACCCGCGGCGCATTaGCTAGTTGGTGAGGTAACGGCTCACCAAGGCAACGATGCGTAGCCGACCTGAGAGGGTGATCGGCCACACTGGGACTGAGACACGGCCCagaCTCCTACGGGAGGCAGCAGTAGGGAATCTTCCACAATGGAcgaAAGTCTGATGGAGCAACGCCGCGTGAGTGAAGAAGGATTTCGGTTCGTAAAACTCTGTTGCAAGGGAagaACAAGTAGCGTagtAACTGGCGCTACCTTGACGGTACCTTGTTAGAAAGCCACGGCTAACTACGTGCCAGCAGCCGCGGTAAtannnagGTGGCAAGCGTTGTCCGGAATTATTGGGCGTAAAGCGCGCGCAGGTGGTTCCTTAnnnCtGATGTGAAagcCCCCGGCTCAACCGGGGAGGGTCATTGgaaactgGGGAACTtg |
| *Variovorax paradoxus*  gGnAtCGCCCTCCTTGCGgTTAAGCTAACTACTTCTGGCAGAACCCGCTCCCATGGTGTGACGGGCGGTGTGTACAAGACCCGGGAACGTATTCACCGTGACATTCTGATCCACGATTACTAGCGATTCCGACTTCACGCAGTCGAGTTGCAGACTGCGATCCGGACTACGACTGGTTTTATGGGATTAGCTCCCCCTCGCGGGTTGGCAACCCTTTGTACCAGCCATTGTATGACGTGTGTAGCCCCACCTATAAGGGCCATGAGGACTTGACGTCATCCCCACCTTCCTCCGGTTTGTCACCGGCAGTCTCATTAGAGTGCCCAACTGAATGTAGCAACTAATGACAAGGGTTGCGCTCGTTGCGGGACTTAACCCAACATCTCACGACACGAGCTGACGACAGCCATGCAGCACCTGTGTTACGGTTCTCTTTCGAGCACTAAGCCATCTCTGGCGAATTCCGTACATGTCAAAGGTGGGTAAGGTTTTTCGCGTTGCATCgAATTAAACCACATCATCCACCGCTTGTGCGGGTCCCCGTCAATTCCTTTGAGTTTCAACCTTGCGGCCGTACTCCCCAGGCGGTCAACTTCACGCGTTAGCTTCGTTACTGAgTCAGTGAAGACCCAACAACCAGTTGACaTCGTTTAGGGCGTGGACTACCAgGGTATCTAATCCTGTTTGCTCCCCACGCTTTCGTGCATGAgCGTCAgTACaGGtCCAGgGGgaTTGCCTTCnccntcggTGTTCCtCcCGCAtnaTCTACgCaTTtCACTGCtACACgCGgaatTCCATCcccnnCTACcGTAcTCt |
| **Fungi** |
| *Clonostachys rosea*  aCAAnCTCcAanCccaTGTGAactACCTACTgTtGCTTCGGcGGgATTGCCCcGGgCGCCTcgtGTGCCCCGgaTCAGGCGCCCGCCTaGgAAACttAaTtcttGTTTTATTTtggAAtcTtCTGagtanTT |
| *Hypocrea muroiana*  TCagAngTCAAGAAganCCTGGAAaTTGTCTGCCACAACTGTAGTAAAGTGCTGGCTGATGAAGTTGGTCTTGTCTTTTCCATGAAGCCTTTGCCTCACTGGATGCTAACCTGAAATCTACCAGAGTGATCCTGAGTTTGTTGCGGCTATCAATACTCGTGATGCAAAACTCCGTTTCAAGCGGGTATGGGCCGTCTGCAAGAAAAAGCGGAGATGCGAGAATGAGGATCGGACAGACAAgAACAAGGACGAAGAGTTTGCGCCTGGcATAaagACTGCGCTCGtcgcaaGCCATGGcGGCTGTGgCnatGtgCnncCTCaAGTGCgAcaGGtTGCCCTTCaaCTGAngGcGGCTTtcna |
| *Papulaspora sepedonioides*  accnTggcTTncAtCAGCGCGTCAgaagatCCTCGaGaTCGTTTGTCACAACTgtAGCAAGGTGTTGGCTGATAGGGTTaGTTGCCTCTTTTTCGCCCTTGGTGGCCCTTGATGGTAAAAAGCAAAGGTTCCCTAGATTGCTAACACTCACTAGTCTGATCCCGACTTTGCTGCGGCCATGAAGATTCGGAATCCAAAGCAACGGTTCGAAGCGGTTCACAGAGTGTGCAAGAAGAAAAATCGTTGCGagAaCGAATCCTTGAAGAAGGAGGAAAAtGagTTTgatCCCGCTganAaGCCCAAGGGTCCCGCcGCCGgtnaCGgaGgCTgngtAAaTGaCcATCCTACcatccgcna |
| *Pseudogymnoascus roseus*  GAgananCAAGAAGCTCCTCGAGAtCGCCTGCCACAACTGTgncAaGATTTTACTTGACAGAGTTAGTTATCCCGCATGGGATGGCATCTTCTTGTAATTGAGTGCTGACATTAGTGATTTTCCTAGAGTAACCCACAGTTCAAAGCTGCCGTCTCGATGCGCGACCCTAAGCGTAGGTTCGAAGCGATCTGGCGTCTGTGCAAGCCAAAGATGATCTGTGACGCCGATATTGCGGTAGATGATGACGAGTTTGCTGCGGATCCGAAGGCAGCTGCgAAGAGGCCAAGCCACGGTGGCTGCGGAAACACACAACCCGAAGTCCGACAGACGGCGTTGCAGCTGTGGGGAACGTGGAAGGTGCCAAAGGATGAGGATaACGAgAACGGACAACCGgAgAanAagCtGAtcaCACcAnAAATGGCGTTGCagGTGTTCCgtaaTATcnCcaCTGaCGAtaTCtACGACCTAtGtCTCancanc |
| *Trichoderma viride*  ttCTTCgAATCTCGCGCGATAtGATGCGGCGAGCCCTCACGAATGGCTTGCTTGAnnnnnnngTTGGCACGAATGATGTCTCCGAGTTTGTAGGTCAAGTCATCTTCGTTACGCATGCCGGTTCCAGTACCATCCATGGAAATAGAAGGGCGGACGGGAGGCGGGGGGACAGGGAGCACGGTGAGAATCATCCATTCTGGACGGGCGTAGTCCGAATTAAGACCCATGTTGACAAGATCCCCTTCAGAGATGCGGCGAAGGATACCGTGGGCCATCTCGGGGGTGATGGGGGCCGTTTCTTTCCTCTTGGGGCCATCCTCCTGAGCTACCTCgAAAGCCGCCTTCAGTTGAAGGGCAACCtGncgnnnttgAnGCTGCACATTGCCACAGCCGCCaTGGCTTGCnAcgAgCGCAgTCTTTATGCCAGGCgCAaACTCTTCGTCCTTGTTCTTGTCTGTCCgATCCTCATTCtCgCAtCTCCncnttTTCttgnagAcggnccatACCCGCTTGAAaCggAgTTTTGCatCacnA |

Sequences were obtained from GATC Biotech (Konstanz, Germany). N, unknown nucleotide; lower case letters indicate uncertain nucleotides.
