# Supplementary material for: Disruption of microbial community composition and identification of plant growth promoting microorganisms after exposure of soil to rapeseed-derived glucosinolates
Source: PLoS One. 2018 Jul 3;13(7):e0200160. doi: 10.1371/journal.pone.0200160 (PMC6029813; doi:10.1371/journal.pone.0200160)
Supplement: S7 Table — Abundances of the microorganisms on genera level as detected by DNA sequencing are presented for the strains analyzed for their PGP properties. (DOCX) [file pone.0200160.s017.docx]

**S7 Table. Abundance of cultivable microorganisms.**

Abundances of the microorganisms on genera level as detected by DNA sequencing are presented for the strains analyzed for their PGP properties.

| Taxonomic Level of identified culturable Microorganisms | Abundance control % | | | | Abundance  RS-EX % | | | |
| --- | --- | --- | --- | --- | --- | --- | --- | --- |
| Time (days) | 7 | 14 | 21 | 28 | 7 | 14 | 21 | 28 |
| *Aminobacter* | 0 | 0 | 0.05 | 0.06 | 0.05 | 0 | 0.05 | 0.13 |
| *Mycobacterium* | 0.3 | 0.2 | 0.15 | 0.2 | 0.1 | 0 | 0 | 0 |
| *Bacillus* | 0.15 | 0.2 | 0.15 | 0.2 | 0.55 | 0.8 | 0.2 | 0.07 |
| *Paenibacillus* | 0 | 0.05 | 0.05 | 0.05 | 0.1 | 0.05 | 0 | 0 |
| *Lysinibacillus* | 0 | 0 | 0 | 0 | 0 | 0 | 0 | 0 |
| *Papulaspora* | 0.4 | 0.55 | 0.4 | 0.37 | 0 | 0 | 0.03 | 0.03 |
| *Trichoderma* | 1 | 0.8 | 0.3 | 0.15 | 0.2 | 0.1 | 0.3 | 0.1 |
